# macroH2A2 antagonizes epigenetic programs of stemness in glioblastoma

**DOI:** 10.1038/s41467-023-38919-2

**Published:** 2023-05-27

**Authors:** Ana Nikolic, Francesca Maule, Anna Bobyn, Katrina Ellestad, Seungil Paik, Sajid A. Marhon, Parinaz Mehdipour, Xueqing Lun, Huey-Miin Chen, Claire Mallard, Alexander J. Hay, Michael J. Johnston, Christopher J. Gafuik, Franz J. Zemp, Yaoqing Shen, Nicoletta Ninkovic, Katalin Osz, Elodie Labit, N. Daniel Berger, Duncan K. Brownsey, John J. Kelly, Jeff Biernaskie, Peter B. Dirks, Darren J. Derksen, Steven J. M. Jones, Donna L. Senger, Jennifer A. Chan, Douglas J. Mahoney, Daniel D. De Carvalho, Marco Gallo

**Affiliations:** 1 Arnie Charbonneau Cancer Institute, Cumming School of Medicine, University of Calgary, Calgary, AB Canada; 2 Alberta Children’s Hospital Research Institute, Cumming School of Medicine, University of Calgary, Calgary, AB Canada; 3 Department of Biochemistry and Molecular Biology, Cumming School of Medicine, University of Calgary, Calgary, AB Canada; 4 Department of Biological Sciences, Faculty of Science, University of Calgary, Calgary, AB Canada; 5 Princess Margaret Cancer Centre, Toronto, ON Canada; 6 Ludwig Institute for Cancer Research, University of Oxford, Oxford, UK; 7 Canada’s Michael Smith Genome Sciences Centre, BC Cancer, Vancouver, BC Canada; 8 Department of Medical Genetics, University of British Columbia, Vancouver, BC Canada; 9 Department of Oncology, Cumming School of Medicine, University of Calgary, Calgary, AB Canada; 10 Department of Compararive Biology and Experimental Medicine, Faculty of Veterinary Medicine, and Hotchkiss Brain Institute, Cumming School of Medicine, University of Calgary, Calgary, AB Canada; 11 Department of Chemistry, Faculty of Science, University of Calgary, Calgary, AB Canada; 12 Department of Clinical Neurosciences, Cumming School of Medicine, University of Calgary, Calgary, AB Canada; 13 Program in Developmental and Stem Cell Biology, Hospital for Sick Children, Toronto, ON Canada; 14 Department of Molecular Genetics, University of Toronto, Toronto, ON Canada; 15 Department of Microbiology, Immunology and Infectious Diseases, Cumming School of Medicine, University of Calgary, Calgary, AB Canada; 16 Department of Medical Biophysics, Faculty of Science, University of Toronto, Toronto, ON Canada

**Keywords:** CNS cancer, Cancer genomics, Cancer models, Cancer stem cells, Mechanisms of disease

## Abstract

Self-renewal is a crucial property of glioblastoma cells that is enabled by the choreographed functions of chromatin regulators and transcription factors. Identifying targetable epigenetic mechanisms of self-renewal could therefore represent an important step toward developing effective treatments for this universally lethal cancer. Here we uncover an epigenetic axis of self-renewal mediated by the histone variant macroH2A2. With omics and functional assays deploying patient-derived in vitro and in vivo models, we show that macroH2A2 shapes chromatin accessibility at enhancer elements to antagonize transcriptional programs of self-renewal. macroH2A2 also sensitizes cells to small molecule-mediated cell death via activation of a viral mimicry response. Consistent with these results, our analyses of clinical cohorts indicate that high transcriptional levels of this histone variant are associated with better prognosis of high-grade glioma patients. Our results reveal a targetable epigenetic mechanism of self-renewal controlled by macroH2A2 and suggest additional treatment approaches for glioblastoma patients.

## Abstract (web summary)

Self-renewing cells play an important role in initiation, progression, and therapy resistance in glioblastoma. Here, the authors identify histone variant macroH2A2 as a regulator of chromatin organisation resulting in the suppression of transcriptional programs of self-renewal in glioblastoma.

## Introduction

Glioblastoma (GBM) is the most common malignant primary brain tumor in adults and has poor prognoses even in cases treated aggressively with gross total resection, radiation, and adjuvant chemoradiotherapy^1^. Several factors are thought to contribute to the aggressiveness of GBM, especially at recurrence: The activation of drug resistance mechanisms^2^, treatment-induced hypermutation^3–5^, a highly immunosuppressive tumor microenvironment^6,7^, and underlying disease characteristics, most notably a high degree of intratumoral heterogeneity^8,9^. Recent studies have shown that the cellular heterogeneity observed in GBM occurs both at the genetic, epigenetic, and transcriptional levels^8–12^. In particular, single-cell studies have highlighted that transcriptional heterogeneity in GBM reflects the co-existence of heterogeneous populations of tumor cells with different functional properties and transcriptional phenotypes resembling different developmental cell lineages^13–20^. This functional interpretation of epigenetic heterogeneity in GBM well aligns with the experimentally-validated concept that GBM includes cell populations with different functional properties, including self-renewal^9,11,21–24^.

GBM self-renewing cells (GSCs) are expandable in culture^25^, can differentiate into non-self-renewing cell types in vitro^26,27^, and are capable of tumor initiation and serial propagation of the tumor in immunocompromised mice in vivo^24^. Studies have shown GSCs are more resistant to the standard of care treatment for GBM than non-self-renewing cells, and they represent a larger fraction of tumor cells at relapse than at diagnosis^28,29^. GSCs, therefore, play crucial roles in tumor growth, therapy resistance, and relapse. However, therapeutic targeting of self-renewing cells is currently an unmet clinical need.

Epigenetic programs that promote self-renewal are maintained by the choreographed function of several chromatin remodelers. These include the histone methyltransferase DOT1L and arginine demethylase JMJD6^23,30^, members of mixed-lineage leukemia (MLL) family^31,32^, and of the polycomb-repressive complexes 1 and 2 (PRC1/2)^33,34^, among others. These chromatin remodelers promote self-renewal by activating transcriptional networks associated with stemness and by repressing differentiation. This can be achieved through the activation of master transcription factors^9,35^ or by modulating the expression of histone variants^10,31,36,37^, which ultimately shape chromatin organization and downstream transcriptional programs.

Histone variants differ from core histones at key amino acids that determine their patterns of incorporation into chromatin and their effects on the transcription of downstream genes^38^. For instance, *MACROH2A1* (previously known as *H2AFY*) and *MACROH2A2* (previously known as *H2AFY2*) encode two closely related variants of core histone H2A. They share only about 60% sequence identity in their histone domain with core histone H2A^39^, and contain two additional domains: A basic linker region with putative DNA binding function, and a “macro” domain with little sequence conservation between the two paralogs^40^. Because of the large size of the macro domain, the substitution of H2A with macroH2A1 or macroH2A2 is predicted to have large effects on chromatin organization^41^. Loss or downregulation of macroH2A1 and macroH2A2 have been described in a number of malignancies, including bladder cancer, melanoma, lung cancer, and gastric cancer^36,42,43^.

MacroH2A1 was first studied for its role in X chromosome inactivation in female mammalian cells^44^. It localizes to large chromatin domains throughout the genome that encompass developmentally regulated genes and regions of imprinting, and it accumulates at senescence-associated heterochromatic foci^45–49^. Both macroH2A1 and macroH2A2 are involved in the repair of double-strand breaks, and knockdown of macroH2A1 impairs DNA double-strand repair^50–53^. MacroH2A variants are modulators of differentiation capacity in developmental models and are incorporated at pluripotency genes during diffrerentiation^54^. Consequently, the knockout of these genes in mouse embryonic stem cells and pluripotent stem cells results in impaired differentiation^55^ and abnormal embryoid body morphology^56^. Knockdown or knockout of macroH2A paralogs increases the reprogramming efficiency of somatic cells into induced pluripotent stem cells^55^. Moreover, mice with knockout for a macroH2A1 splice variant (macroH2A1.2) have abnormalities in neural progenitor cell differentiation^57^ and knockdown of macroH2A1 leads to memory impairment^58^. Collectively, these results suggest that macroH2A variants might be involved in the repression of stemness properties, including self-renewal. The molecular mechanisms exploited by macroH2A paralogs in modulating stemness and self-renewal are not currently understood, especially in the context of cancer. Given the current inability to target self-renewing cells in most cancer types, dissection of these mechanisms could be important to identify new treatment options for GBM and other malignancies characterized by intratumoral functional heterogeneity.

Here we deploy epigenomic and functional assays to investigate the mechanisms utilized by macroH2A2 to modulate self-renewal programs in GBM. In this work, we show that macroH2A2 is a negative regulator of self-renewal in glioblastoma stem cells, and repression of macroH2A2 abrogates cellular transitions from proneural to mesenchymal/astrocytic cellular states.

## Results

### Low expression of *MACROH2A2* is a negative prognostic factor for GBM patients

**Fig. 1:**
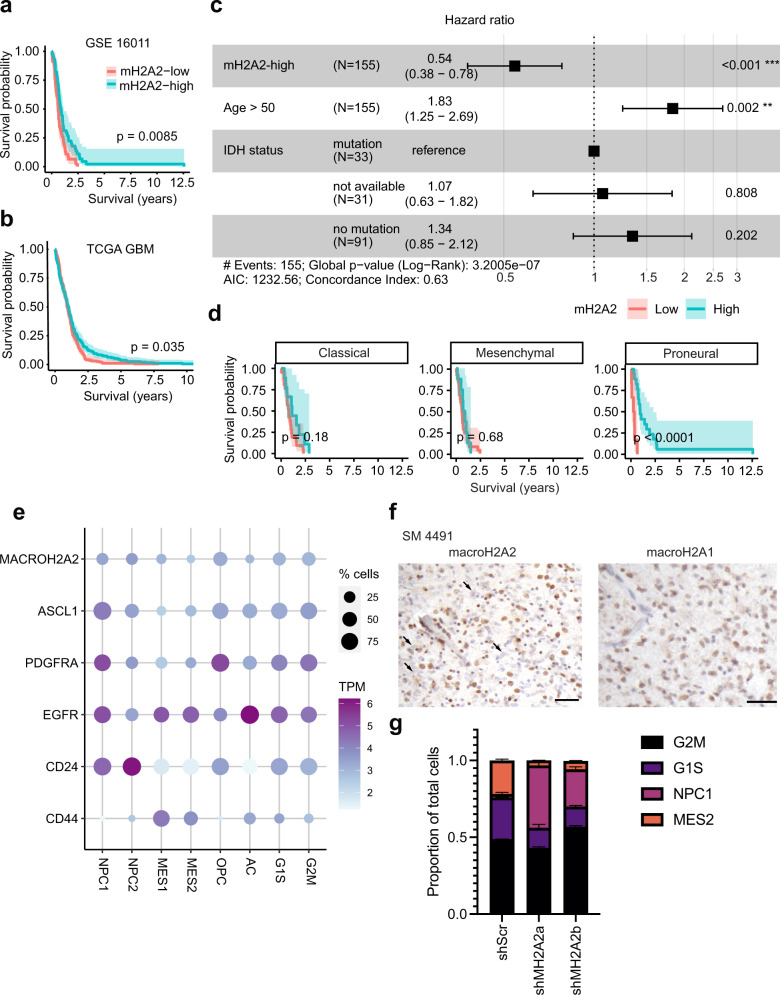
Low transcription levels of *MACROH2A2* contribute to GBM aggressiveness. **a**, **b** Kaplan–Meier survival analysis of **a** all high-grade gliomas in TCGA GBM cohort^60^ (*n* = 336) and **b** adult *IDH*-wildtype glioblastoma patients (GSE16011^61^; *n* = 86) based on *MACROH2A2* (mH2A2) transcription levels. mH2A2*-*low and -high groups were determined by median gene expression. The shaded region represents a 95% confidence interval. *P* value was obtained by log-rank test. **c** Hazard ratios for macroH2A2 expression in GSE16011 (*n* = 155) in a multivariate Cox regression model adjusting for other factors relevant for glioblastoma (age, IDH mutation status). Error bars represent 95% confidence intervals. **d** Kaplan–Meier survival analysis of adult IDH-wildtype glioblastoma patients (*n* = 86) separated by transcriptional subtype (GSE16011). The shaded region represents a 95% confidence interval. *P* values obtained by log-rank test. **e** Kaplan–Meier survival analysis of adult IDH-wildtype primary glioblastoma patients with the proneural transcriptional subtype (GLASS consortium; *n* = 17). The shaded region represents a 95% confidence interval. *P* values obtained by log-rank test. **f** Expression of *MACROH2A2* (mH2A2) in the scRNA-seq GBM dataset by ref. ^8^ plotted on a 2D state diagram. **g** CIBERSORTx decomposition of RNA-seq datasets from control and knockdown G523 cells (three biological replicates per condition); (*p* = 0.021 (NPC shScr vs shMH2A2a, *p* = 0.01823 (MES shScr vs shMH2A2a); NPC *p* = 0.0075 shScr vs shMH2A2b; MES *p* = 0.0116 shScr vs shMH2A2b) [*p* values: two-tailed unpaired T-test with Welch’s correction]. Error bars represent standard deviation. **h** Immunohistochemistry of macroH2A2 and macroH2A1 in a primary patient glioblastoma specimen (scale bar: 50 µ). The experiment was repeated on three independent primary clinical samples.

We embarked on a systematic analysis to identify genes encoding histone variants that may be involved in adult high-grade glioma biology. Our approach consisted in identifying histone variant genes whose transcriptional levels could stratify high-grade glioma patients based on overall survival in two cohorts: One collected by The Cancer Genome Atlas (TCGA)^59,60^ and one described by ref. ^61^. Median transcriptional levels were used to classify patients as high- or low-expressors for each gene. Transcriptional data were available for 9 histone variant genes in the Gravendeel cohort and for 11 genes in the TCGA cohort. Only *MACROH2A2*, the gene encoding the histone variant macroH2A2, produced statistically significant patient stratification in both cohorts (Figs. S1a–h, S2a–j). We found that low levels of *MACROH2A2* transcription were associated with shorter overall survival in the high-grade glioma cohort collected by TCGA (log-rank *p* = 0.035; Fig. 1a) and in IDH-wildtype GBM patients in the Gravendeel cohort (log-rank *p* = 0.0085; Fig. 1b). To better tease out potential confounding effects of IDH status in the TCGA cohort, we stratified tumors by CpG island methylator phenotype (CIMP) status, which is tightly associated with IDH mutations^62,63^. *MACROH2A2* transcriptional levels did not stratify patients with IDH-mutant gliomas in the Gravendeel cohort or CIMP-positive tumors in the TCGA cohort (Fig S3a–b). In CIMP-negative tumors, however, there was a trend towards increased survival with higher macroH2A2 levels, but this was not significant (Fig S3c). CIMP-negative patients who received combined chemoradiotherapy showed a significant survival benefit with higher *MACROH2A2* expression (*p* = 0.03; Fig. S3d). In the Gravendeel dataset, a multivariate Cox regression model showed that increased *MACROH2A2* expression had prognostic significance (hazard ratio: 0.54 [0.38 – 0.78]; Fig. 1c), even when adjusted for known prognostic factors such as IDH mutation status, age, and treatment status. In the TCGA cohort, the survival benefit associated with high *MACROH2A2* transcription was also observed in patients with recurrent disease, and recurrent tumors had significantly higher levels of *MACROH2A2* expression than tumors at diagnosis (Fig S3k, l). On the other hand, transcription levels of the paralog *MACROH2A1* had no prognostic significance in an either patient cohort (Figs. S1c, S2c) and appeared consistently high in all tumors (Fig. S3h). When Verhaak molecular subgroups^59^ were considered in the ref. ^61^ cohort of IDH-wildtype GBM, the survival benefit of higher *MACROH2A2* expression was exclusive to proneural tumors (*p* < 0.0001, log-rank test) in the Gravendeel cohort (Fig. 1d) and trended towards significance in the GLASS consortium^64^ (Fig. 1e). No patient stratification was observed in patients with classical or mesenchymal tumors (Fig. 1d).

The Verhaak classification of GBM mentioned above is based on bulk RNA-seq data. Therefore next, we investigated *MACROH2A2* expression in the context of transcriptional states recently identified through single-cell RNA-seq (scRNA-seq) analyses. In the dataset generated by Neftel and colleagues^8^, *MACROH2A2* expression is enriched in cells with transcriptional modules reminiscent of neural progenitor cells and oligodendrocyte progenitor cells (NPC1, NPC2, and OPC), and was markedly lower in mesenchymal (MES1 and MES2) and astrocytic (AC) subtypes (Fig. 1f, g and Fig. S4a). *MACROH2A2* expression is also enriched in cycling cells (Fig. S4a). However, when cycling cells are considered separately, transcription of this gene is still significantly enriched in the NPC1 (*p* < 2.22e-16; *p* = 2.5e-8), NPC2 (*p* = 1.1e-8; *p* < 2.22e-16), and OPC (*p* < 2.2e-16; *p* < 2.2e-16) compartments compared to MES1 and MES2, respectively (Fig. S4a). In a second scRNA-seq dataset generated by ref. ^16^, *MACROH2A2* is expressed at higher levels in cells with high scores for the developmental subtype (Fig. S4b, c), which largely corresponds to NPC subtypes in the Neftel nomenclature (Fig. 1e). This parallels the expression patterns of *MACROH2A2* in human and mouse brain^65,66^, where it is enriched in proliferating fetal progenitor-like cell types, including fetal astrocytes in humans, and SOX4-positive neural progenitors in mouse (Fig. S3i, j). Interestingly, RNA-seq datasets of GBM from regionally sampled mouse xenografts^67^ and human tumors^68^ show increased *MACROH2A2* expression in the tumor core, with lower levels at the margin or infiltrating edge (*p* = 0.024 for GSE139261 and *p* = 0.0094 GSE117891; Wilcoxon test) (Fig. S4d, e). Collectively, our analyses of patient cohorts (bulk data) and single-cell transcriptomes generated from different groups consistently indicate that the transcription of *MACROH2A2* is enriched in glioma cells with proneural or NPC-like states, and reduced at the infiltrating edge of the tumor.

We next followed up on this observation and investigated whether modulating macroH2A2 expression can alter the transcriptional flavor of GBM cells. We generated doxycycline-inducible *MACROH2A2* knockdown models (Fig. S5b–g) in three patient-derived cultures (G523, GSC2, and GSC3). These *MACROH2A2* knockdown constructs were specific to this H2A variant and showed no effect on protein levels of the related variant macroH2A1 (Fig. S5d). All three parental cultures showed a distribution of both mesenchymal and neurodevelopmental-like cells (Fig. S5a) and shared typical molecular and genetic features of IDH-wildtype GBM (Supplementary Table 1). When we examined the effects of knockdown on the relative proportion of cells in the different transcriptional subtypes, we noted a marked increase in NPC-type cells (*p* = 0.021 for shMH2A2a; *p* = 0.0075 for shMH2A2b; two-tailed unpaired *T*-test) and reduction in MES-type cells (*p* = 0.018 for shMH2A2a; *p* = 0.0116 for shMH2A2b; two-tailed unpaired *T*-test) upon knockdown of macroH2A2 (Fig. 1g). This suggested that knockdown of macroH2A2 was altering the cell state equilibrium of our GBM primary cultures to favor NPC-like states. Collectively, our results point to a role for macroH2A2 in modulating the balance between proneural/NPC/OPC/developmental-like vs MES/AC transcriptional cell states in GBM.

We next assessed the expression of macroH2A2 at the protein level in primary patient tumors. Immunohistochemical staining of primary patient specimens for macroH2A2 showed a mosaic pattern, with some cells having relatively higher levels of this protein and others having lower levels (Fig. 1h). Expression of the close paralog macroH2A1 was more homogeneous in all GBM cells (Fig. S3c). These results broadly confirm transcriptional data (Fig. S2d) and suggest that expression of macroH2A2 is intratumorally heterogeneous in GBM.

### macroH2A2 antagonizes self-renewal in GBM

**Fig. 2:**
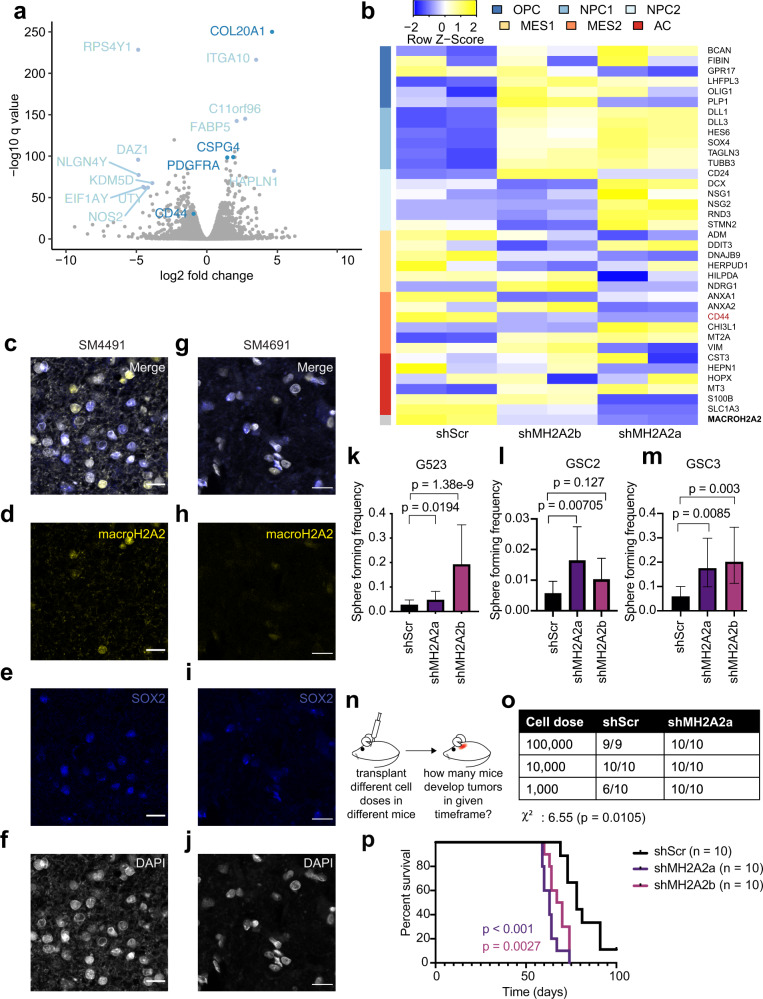
macroH2A2 antagonizes self-renewal in GBM and is suppressed in stem-like cells. **a** Volcano plot highlighting differentially expressed genes after 7 days of *MACROH2A2*/macroH2A2 knockdown in G523 cells. **b** RNA-seq was used to determine transcriptional levels of the top genes of the state metamodules from ref. ^8^ at 7 days following *MACROH2A2*/macroH2A2 knockdown. Two biological replicates were used per condition. **c**–**j** Confocal microscopy images of macroH2A2 and SOX2 in two primary patient tumors (SM4491 (**c**–**f**) and SM4691 (**g–j**)). Scale bars: 20 µ. The experiment was performed once on three independent clinical samples. **k**–**m** Limiting dilution assay results after 14 days of doxycycline induction in G523 glioblastoma cells (**k**), GSC2 (**l**), and GSC3 (**m**). The center point represents a calculated estimate of sphere formation. *P* value was determined by the Chi-square test with the tool ELDA (see Methods). Error bars: 95% confidence interval. Statistics from six technical replicates; the experiment was repeated three times. **n** Schematic of in vivo limiting dilution assay. **o** Overview of in vivo limiting dilution assay results. Mice were transplanted orthotopically with either shScr or shMH2A2a-transduced GSCs. *P* value and chi-square value obtained by Chi-square test. **p** Orthotopic xenograft experiments to assess the effects of *MACROH2A2* knockdown on survival of transplanted mice. Patient-derived GSCs carrying either scrambled control shRNA constructs (shScr; *n* = 10; one mouse censored) or independent shRNAs targeting *MACROH2A2* (shMH2A2a/b; *n* = 10 mice per group) were transplanted orthotopically in immunocompromised mice. *P* values were calculated with the log-rank test.

Self-renewal, or self-replication, is a key property of a population of cells that propagate tumor growth^69^. It was shown that the fraction of GBM cells that can self-renew, as well as transcriptional signatures associated with self-renewal, are negative prognostic factors for this brain cancer^70^. Given the significance of self-renewal in GBM, we investigated whether macroH2A2 plays a role in modulating this crucial functional property. First, we analyzed RNA-sequencing data we generated using control and knockdown GBM cells (see above). Volcano plots illustrated that some of the most upregulated genes upon *MACROH2A2* knockdown are highly expressed in OPCs, including *COL20A1*, *CSPG4*, and *PDGFRA* (Fig. 2a)^71^. We also identified a marked increase in marker genes associated with the Neftel et al.^8^ OPC, NPC1, and NPC2 signatures and reductions in MES and AC signature genes, including *CD44* (Fig. 2a, b). These data suggest a role for macroH2A2 in repressing gene expression signatures associated with self-renewal and the oligodendrocytic lineage. To further validate this observation, we went back to our primary patient tumors, and performed multiplex fluorescence microscopy for macroH2A2 and SOX2 (Fig. 2c–j). This imaging revealed that most of the primary tumor cells that were SOX2-positive had very low levels of macroH2A2, whereas macroH2A2 signals was strongest in the adjacent SOX2-negative cells. Both of these observations suggested that macroH2A2 levels are reduced in the self-renewing population of GBM cells. We, therefore, tested this hypothesis at the functional level.

The frequency of self-renewing cells in a population can be estimated with in vitro limiting dilution assays (LDAs), an established method that uses sphere-forming frequency as a measure of self-renewal^72^. We used our doxycycline-inducible *MACROH2A2* knockdown models (Fig. 2a, b and Fig. S5a–d) for in vitro LDAs in three different primary patient-derived cultures (G523, GSC2, and GSC3). Knocking down *MACROH2A2* resulted in increased self-renewal in all GSC models (Fig. 2k–m and Fig S5e–g). Moreover, while macroH2A2 knockdown increased the expression of stem-like and OPC-like genes such as *PDGFRA* and *OLIG2*, it did not impair differentiation (Fig. S5h). In order to cross-check these results with an independent experimental system, we decided to overexpress *MACROH2A2* using a fusion protein composed of catalytically dead Cas9 (dCas9) and the transcriptional activator VPR (dCas9-VPR system^73^; Fig. S5i). We generated stable GSC lines bearing a PiggyBac-based dCas9-VPR construct to create a stable inducible overexpression model (Fig. S5i). We then transfected this GSC line with a pool of five single guide RNAs (sgRNAs) targeting *MACROH2A2*. RT-qPCR showed that this experimental system resulted in 10-fold overexpression of *MACROH2A2* over control cells transfected with scrambled sgRNAs (sgScr), with no effects on transcription levels of the close paralog *MACROH2A1* (Fig. S5j, k). We, therefore, employed these *MACROH2A2*-specific overexpression models in in vitro LDAs, which showed that *MACROH2A2* overexpression causes a ~50% reduction in sphere-forming frequency (Fig. S5k). Therefore, our knockdown and overexpression systems concordantly show an antagonistic effect of macroH2A2 on self-renewal in patient-derived GBM cultures.

To further validate these findings, we performed in vivo LDAs. We transplanted GSCs carrying our dox-inducible stable shMH2A2 system or shScr controls into the forebrains of NSG mice (Fig. 2n). We transplanted ten mice at each cell dose (100,000, 10,000, and 1000 cells) for knockdown and control cells. At the lowest dose, ten out of ten mice transplanted with *MACROH2A2*-knockdown cells developed tumors, whereas only six out of ten control mice did (Fig. 2o), representing a significant difference in engraftment potential between GBM cells with *MACROH2A2* knockdown and control cells (*χ*^2^ *p* = 0.0105). Our in vivo and in vitro LDA experiments, therefore, support an antagonistic role of macroH2A2 on the self-renewal of GBM cells.

### macroH2A2 modulates GBM cell state equilibrium in vivo and in vitro

**Fig. 3:**
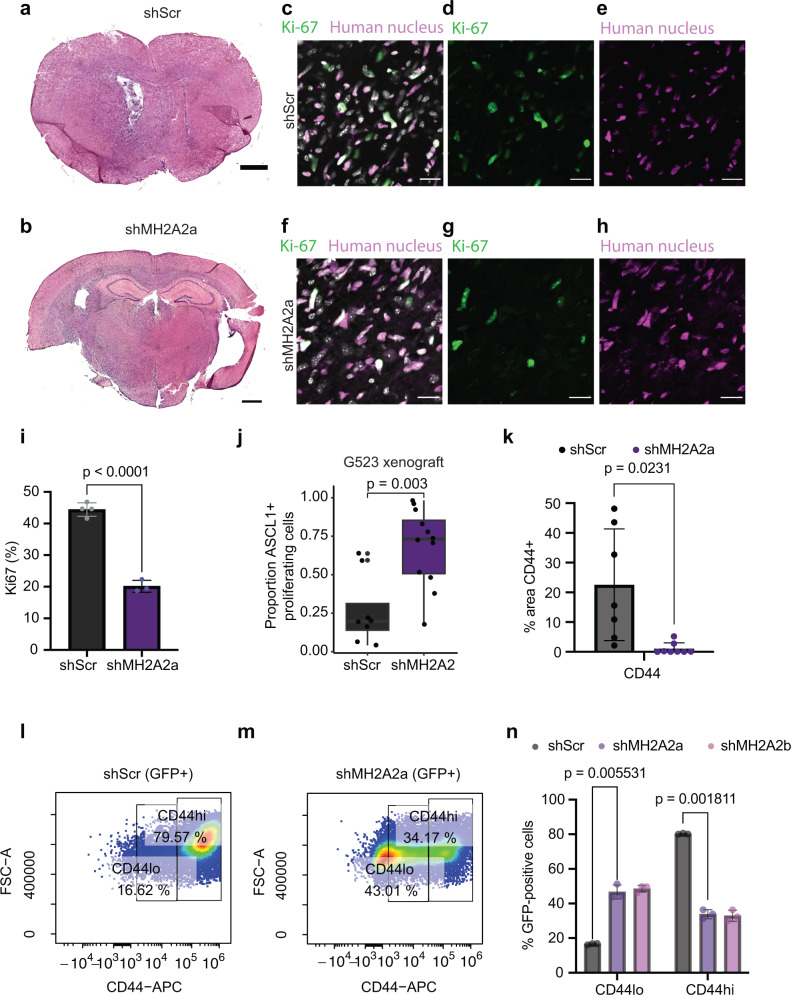
macroH2A2 knockdown leads to enhanced proneural phenotypes in vivo and inhibits CD44-positive cell states. **a**, **b** Representative whole-mount hematoxylin-eosin images of mouse tumors. Scale bar: 1 mm. The experiment was performed once on three mouse tumors per condition. **c**–**h** Confocal microscopy images of Ki-67 and human nucleus staining from scramble [merge (**c**), Ki-67 (**d**), and human nucleus (**e**)] and shMH2A2 knockdown animals [merge (**f**), Ki-67 (**g**), and human nucleus (**h**)] Scale bar 20 mm. **i** Quantification of human Ki-67 positive cells in control versus knockdown mice (*p* value: two-tailed unpaired *T*-test); quantification performed over three 10x fields and repeated in two animals). Error bars represent standard deviation. **j** Quantification of ASCL1-Ki-67 immunohistochemistry in G523 xenograft mice, across *n* = 8 and *n* = 12 independent low-power fields in *n* = 2 distinct animals. The experiment was repeated twice. *P* value: two-tailed unpaired *T*-test with Welch’s correction. The boxplot line represents the median, hinges at 25th and 75th percentiles, and whiskers at 1.5 × IQR. **k** Quantification of CD44 signal in mouse xenografts (*p* = 0.0231; two-tailed unpaired *T*-test); experiment performed in two animals and quantified over at least seven 10x fields. Error bars represent standard deviation. **l**–**n** Flow cytometric analysis of CD44 signal in G523 cells; representative scatterplots of control (**l**) and knockdown (**m**) along with quantification (**n**) performed with three biological replicates (*p* versus shMH2A2a 0.005531 and 0.001811 versus control for CD44-low and CD44-high cells; two-tailed two-tailed unpaired *T*-test with Welch’s correction). Error bars represent standard deviation.

We then decided to further examine the effect of macroH2A2 knockdown on the survival of mice implanted with orthotopic xenografts (Fig. 2p), and found that animals implanted with macroH2A2 knockdown cells became symptomatic sooner and had shorter survival (*p* < 0.001 for shMH2A2a, *p* = 0.0027 for shMH2A2b; log-rank test). Histologic examination of the tumors showed markedly infiltrative lesions in both conditions (Fig. 3a, b) and confirmed that macroH2A2 knockdown was maintained during the in vivo experiment (Fig. S6a–h). To determine if the shortened mouse survival was due to increased fractions of cycling cells, we performed Ki-67 staining of our xenografts (Fig. 3c–i), which surprisingly found that a smaller proportion of tumor cells was cycling in the knockdown tumors compared to the control (*p* < 0.0001; unpaired two-tailed *T*-test). To see if the proliferating cells were NPC-like, we performed co-staining of Ki-67 and ASCL1, and this showed an increased proportion of ASCL1 positive cycling cells in the shMH2A2 mouse tumors (*p* = 0.003; Fig. 3j). This observation was corroborated in our in vitro cell culture system (Fig. S6i–t). This result suggests that the characteristics of the cycling tumor cell population are distinct between the knockdown and control cells. We then went back to our in vitro models and performed flow cytometric cell cycle analysis gating on GFP-positive induced cells, to characterize any differences in proliferation between our control and knockdown cells, and found slight increases in S and G2M cells with a slight reduction of the proportion of G1S cells (Fig. S7e–g). Overall, macroH2A2 has therefore, small effects on cell cycling properties both in vivo and in vitro.

Subsequently, we performed immunofluorescence experiments for the stemness and OPC-associated transcription factor OLIG2, which we found to be upregulated upon *MACROH2A2* knockdown. We noted significant increases in the proportion of cells positive for this marker in our knockdown tumors versus control (Fig. S8a–h). These results confirm our RNA-seq data shown above. As we had noted that CD44, a marker of mesenchymal-type cells, was reduced in our knockdown cells in vitro, we characterized this marker in our xenografts. We performed immunofluorescence for CD44 using orthotopic xenografts from mice transplanted with either control or knockdown GSCs. We found that the control tumors were heterogenous, with many areas showing clusters of human tumor cells with CD44 immunopositivity (Fig. 3j and Fig. S8i–l). On the contrary, knockdown tumors were largely devoid of CD44^+^ cells (Fig. 3j and Fig. S8m–p). To see if we could note a similar effect in vitro, we analyzed knockdown cells induced for one week versus control cells by flow cytometry for CD44 (Fig. 3k–m), which showed a marked reduction in CD44 signal upon *MACROH2A2* knockdown (*p* = 0.001811 against shScr). These data suggest that macroH2A2 is involved in mediating state transitions in GSCs and that knockdown of macroH2A2 pushes cells away from MES states and toward more pronounced NPC/OPC transcriptional states.

### MacroH2A2 maintains chromatin organization at developmentally regulated genes

**Fig. 4:**
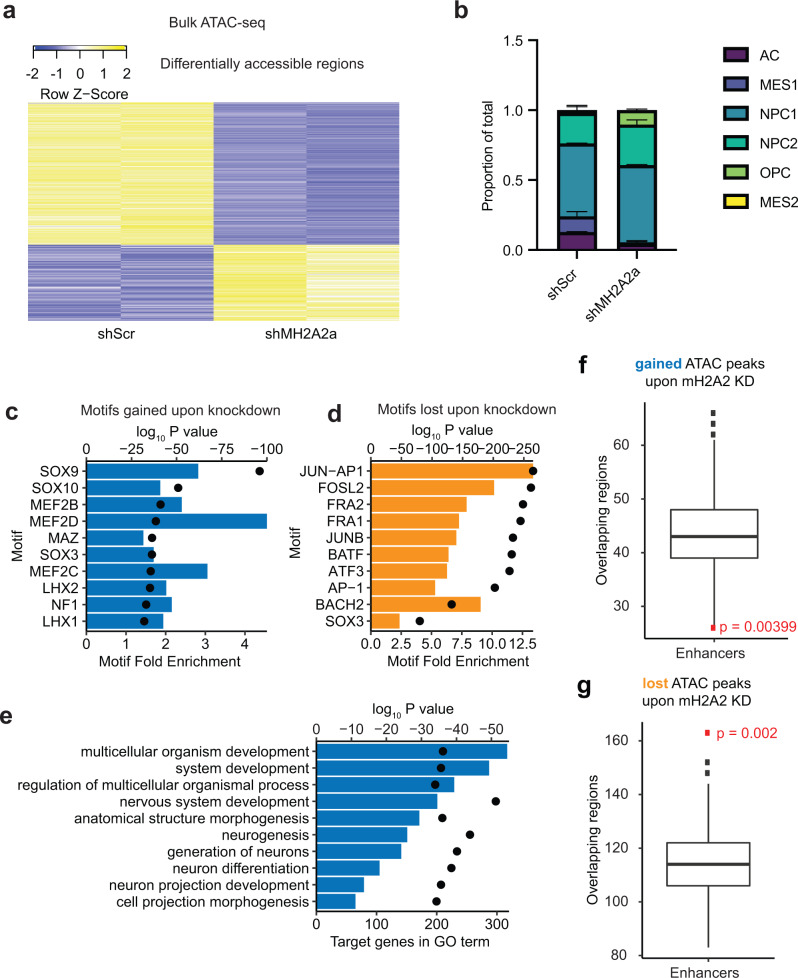
MacroH2A2 contributes to both compacted and accessible chromatin at neurodevelopmental genes in GBM. **a** Heatmap of differentially accessible regions (*n* = 270) in two biological replicates of *MACROH2A2*/macroH2A2 knockdown versus control cells. **b** CIBERSORTx deconvolution of estimated cell states in control versus knockdown cells, averaged across two biological replicates (*p* value 0.02 for NPC1, 0.03 for OPC; 0.14 for MES2; 0.12 for AC; 0.28 for NPC2). *P* values by *T*-test with Welch’s correction. **c** Top ten motifs enriched in peaks gained upon macroH2A2 knockdown (*P* value by hypergeometric test). **d** Top ten motifs enriched in peaks lost upon macroH2A2 knockdown (*P* value by hypergeometric test). **e** Top process terms resulting from Gene Ontology term analysis of peaks gained upon macroH2A2 knockdown (*P* value by hypergeometric test). **f**, **g** Permutation analysis of accessible regions gained (**f**) and lost (**g**) upon *MACROH2A2*/macroH2A2 knockdown at enhancer elements genome-wide. Results were obtained from *n* = 500 permutations of *n* = 94 (**f**) and *n* = 176 (**g**) independent genomic regions. The boxplot line represents the median, hinges at 25th and 75th percentiles, and whiskers at 1.5 × IQR. *P* value by hypergeometric test.

MacroH2A variants have been mostly studied in the context of their roles in chromatin compaction, including X inactivation^44^. However, there is emerging evidence that macroH2A variants can also be found at sites of open chromatin^74,75^. To disambiguate the function of macroH2A2 in epigenetic programs of GBM, we performed the sequencing-based assay for transposase-accessible chromatin (ATAC-seq)^76,77^ in our patient-derived dox-inducible knockdown models. ATAC-seq was performed on two biological replicates for both shScr control cells and *MACROH2A2* knockdown cells. We found that *MACROH2A2* knockdown caused both gains and losses of chromatin accessibility (Fig. 4a and Supplementary TabData 1), although losses were more frequent (*n* = 94 peaks gained; *n* = 176 peaks lost). To better understand the effect on cell states, we generated a cell-type reference for ATAC-seq using previously published data^78^ and applied deconvolution to estimate the proportions of different cell states in our control and knockdown cells (Fig. 4b). This analysis revealed that knockdown led to an increased frequency of cells in the NPC1 and OPC chromatin states (*p* = 0.02 and 0.03, respectively; two-tailed unpaired *T*-test), and a trend towards lower numbers of cells in MES1 and AC states. These ATAC data support the notion that macroH2A2 contributes to the modulation of transitions between distinct chromatin states in GBM.

HOMER motif analysis^79^ revealed that chromatin accessibility changes occur at sites with significant enrichment for DNA recognition motifs of transcription factors associated with neurodevelopment. The top enriched motifs in areas that gained accessibility were SOX9 and SOX10, which are associated with neural crest development and oligodendrocyte differentiation^80–82^, as well as LHX1 and LHX2, both of which are established proneural transcription factors^83^ (Fig. 4c). In contrast, sites that lost chromatin accessibility in the knockdown cells were enriched for AP-1 family motifs, including JUN-AP-1, FOSL2, JUNB, ATF3, and AP-1 (Fig. 4d). This is also in agreement with a decrease in astroglial phenotypes and promotion of proneural and OPC-like states in *MACROH2A2* knockdown cells. Gene ontology analysis of genes that gained chromatin accessibility upon *MACROH2A2* knockdown identified significant enrichment for genes involved in neurodevelopmental pathways, including the terms “nervous system development” and “neurogenesis” (Fig. 4e). Genes that lost chromatin accessibility were enriched in developmental terms as well, but also showed enrichment for cell differentiation and cellular movement/migration (Fig. S8b). These findings, together with the developmental regulation of *MACROH2A2* we demonstrated above (Fig. S3i, j), implicate macroH2A2 in fine-tuning brain-specific epigenetic and transcriptional programs of self-renewal by modulating chromatin organization and state transitions.

Next, we investigated whether macroH2A2 levels had a greater impact on global chromatin architecture, as we have reported for the histone variant H3.3^10^, compared to its effects on the local chromatin environment. We have previously shown that ATAC changepoint analysis provides a measure of global changes in chromatin architecture^10^. We performed changepoint analysis using our ATAC-seq datasets generated with shScr and shMH2A2 GBM cells, and we did not observe large structural differences with the exception of chromosomes 3, 5, and 10 (Fig. S9a). We conclude that macroH2A2 has dual roles in maintaining compacted and accessible chromatin in regional contexts without causing large-scale chromatin reorganization. We therefore decided to further investigate the potential roles of macroH2A2 in shaping the local chromatin environment.

### macroH2A2 modulates enhancer function in GSCs

**Fig. 5:**
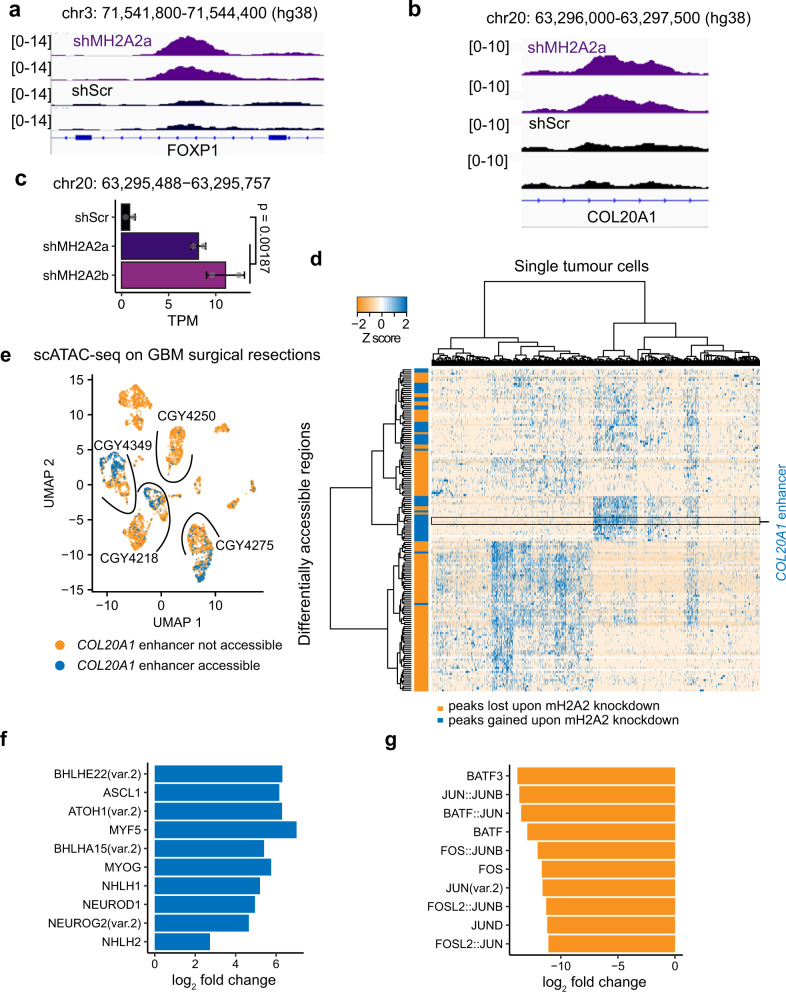
macroH2A2 represses enhancer elements linked to neurodevelopmental genes. **a**, **b** Examples of ATAC-seq enhancer peaks gained upon knockdown of *MACROH2A2*/macroH2A2. **c** Expression of eRNA at the *COL20A1* locus shown in (**b**) in control versus knockdown cells. Two biological replicates were used to generate RNA-seq libraries. *P* value was obtained by a two-tailed unpaired *T*-test with Welch’s correction. **d** Differentially accessible chromatin regions identified upon *MACROH2A2*/macroH2A2 knockdown in a scATAC-seq sample from four primary glioblastoma resections. The horizontal axis represents individual cells in the specimens, with differentially accessible regions listed along the Y axis. **e** Accessibility at the *COL20A1* enhancer in a scATAC-seq dataset generated from four primary GBM surgical specimens. Samples are separated by arcs. **f** Top positively enriched motifs at *COL20A1* accessible cells in primary glioblastoma resections. **g** Top negatively enriched motifs at *COL20A1* accessible cells in primary glioblastoma resections.

There is very little known about the effects of macroH2A2 on chromatin organization and transcriptional control, and this is particularly true in GBM. We initially hypothesized that macroH2A2 might exert its effects on transcription by modulating chromatin accessibility at gene bodies. Surprisingly, permutation analyses revealed a clear depletion of both gained and lost ATAC peaks in gene bodies and their promoters upon *MACROH2A2* knockdown (*p* = 0.002, Fig. S9c–j). On the other hand, ATAC peaks that were gained upon knockdown showed a slight but significant depletion (*p* = 0.0039) at enhancer elements (Fig. 4f), while those lost upon *MACROH2A2* knockdown were significantly over-represented at enhancer elements (*p* = 0.002; Fig. 4g). Specific peaks gained upon knockdown were identified at putative enhancer regions associated with developmental genes, including *FOXP1* and the oligodendrocyte lineage gene *COL20A1* (Fig. 5a, b).

Active enhancers are transcribed by RNA polymerase II, resulting in the production of enhancer RNA (eRNA)^84^. eRNA levels often closely track the transcription levels of their associated genes^85,86^. We reasoned that if macroH2A2 affects the function of enhancer elements, changes in enhancer activity associated with the depletion of macroH2A2 should result in alterations in eRNA transcription levels. Analysis of RNA-seq data from control and *MACROH2A2* knockdown GBM cells showed significant differential transcription of 33 distinct eRNA transcripts, most showing an increased expression in the knockdown cells (Fig. S10a–e). Interestingly, we observed a significant transcriptional increase of the eRNA at the *COL20A1* enhancer locus in our knockdown cells (Fig. 5c), suggesting that macroH2A2 may play a role in repressing this regulatory region. These data are consistent with the effects of *MACROH2A2* knockdown on *COL20A1* transcription, and suggest a mechanism by which macroH2A2 represses this gene associated with the oligodendrocytic lineage by compacting the chromatin at its cognate enhancer element.

To further validate the differentially accessible regions we identified in our knockdown cells, we analysed single-cell ATAC-seq (scATAC-seq) we had previously generated for four adult GBM primary patient samples^78^. Adult GBM resections showed accessibility in the majority of differentially accessible peaks gained and lost upon *MACROH2A2* knockdown, confirming the relevance of the results generated with our patient-derived models. Gained and lost peaks were accessible in two distinct clusters of cells, suggesting that they define populations of cells characterized by distinct chromatin states (Fig. 5d). The *COL20A1* enhancer was also identified in the primary tumor specimens, and its accessibility was restricted to a single cluster of tumor cells in three out of four primary samples profiled by scATAC-seq (Fig. 5e). Moreover, we performed differential motif enrichment analysis using our GBM surgical resections profiled by scATAC-seq. We found that primary tumor cells with increased accessibility at the *COL20A1* enhancer had enrichment for recognition motifs for proneural transcription factors such as ASCL1, NEUROD1, and NEUROG2 (Fig. 5f), whereas cells with decreased accessibility at the *COL20A1* enhancer had depletion of DNA binding motifs associated with AP-1 family transcription factors such as FOS and JUND (Fig. 5g). These results using surgical specimens, therefore, corroborate our observations with our patient-derived GSC models, supporting the linkage of these two states to a neurodevelopmental and mesenchymal-like phenotype. Altogether, our data show that *MACROH2A2* has an important role in mediating enhancer accessibility in GBM cells, and appears to modulate a regulatory network driving GSC cell identity.

**Fig. 6:**
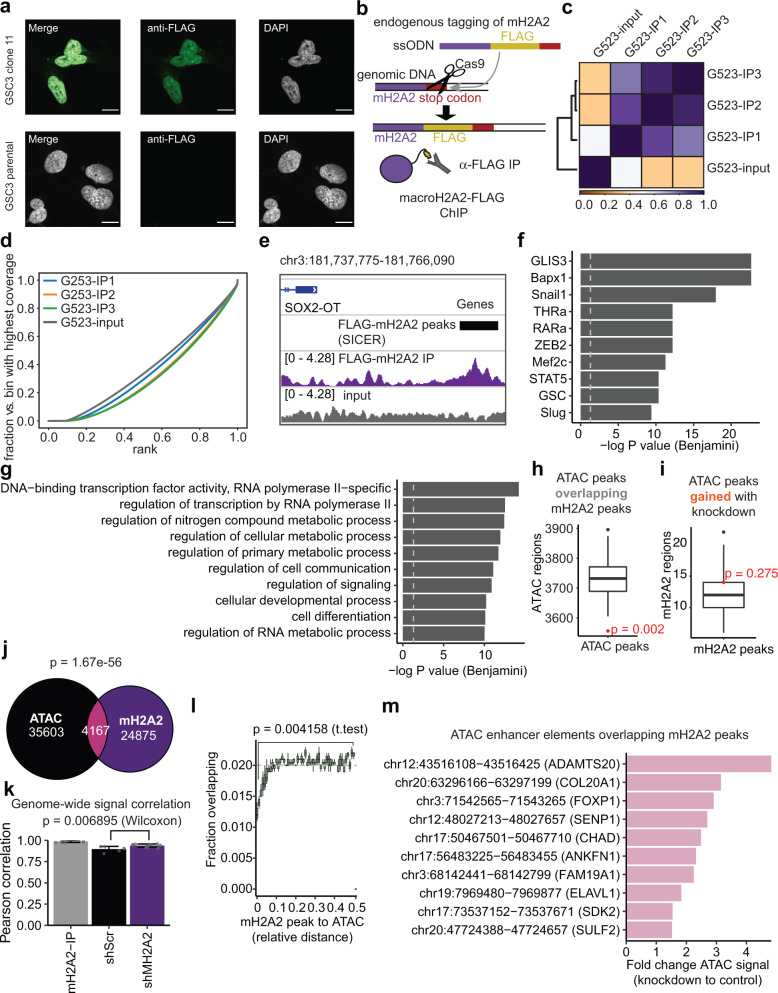
Characterization of chromatin binding patterns of macroH2A2 in glioblastoma stem cells. **a** Immunofluorescence microscopy of FLAG-tagged GSC3 tumor cells versus control. Scale bar: 10 mm. The experiment was performed twice on two independent FLAG-tagged clones. **b** Overview of endogenous tagging strategy. ssODN single-stranded oligodeoxynucleotide template. **c** Heatmap of signal correlation between FLAG-macroH2A2-IP samples and control. **d** Fingerprint plot of FLAG-macroH2A2 ChIP samples versus control. **e** Example of a peak call and associated signal track. **f** Top ten motifs associated with ChIP-seq macroH2A2 peaks. *P* value: hypergeometric test with Benjamini correction. **g** Top Gene Ontology terms associated with macroH2A2 peaks. *P* value: hypergeometric test with Benjamini correction. **h** Permutation analysis of macroH2A2 peaks examining overlap with ATAC peaks (*n* = 500 permutations; *P* value by hypergeometric test). **i** Permutation analysis of ATAC-seq peaks that gain accessibility in knockdown cells overlapping with macroH2A2 peaks. (*n* = 500 permutations; *P* value by hypergeometric test). The boxplot line represents the median, hinges at 25th and 75th percentiles, and whiskers at 1.5 × IQR. **j** Venn diagram comparing macroH2A2 peaks, ATAC-seq peaks, and their overlap (*p* value—expected overlap by Fisher’s test). **k** Pearson correlation of signal across the entire genome between macroH2A2-IP samples and control and knockdown ATAC-seq samples. Pairwise comparisons across *n* = 3 biological ChIP replicates and *n* = 2 biological ATAC replicates. Error bars represent standard deviation. *P* value calculated by Wilcoxon test. **l** Relative distance plot showing the fraction of overlaps versus expected for macroH2A2 peaks compared to ATAC-seq peaks. Each point represents an average of *n* = 3 biological replicates of ChIP. Error bars represent standard deviation. *P* value by unpaired *T*-test with Welch’s correction. **m** Top 10 ATAC-seq enhancer elements which overlap macroH2A2 peaks sorted by differential accessibility.

As our ATAC-seq experiments indirectly suggested a role for macroH2A2 in mediating enhancer function and cellular state identity, we decided to profile this mark by chromatin immunoprecipitation with sequencing (ChIP-seq). We were not able to identify commercially-available antibodies that met our standards for ChIP-seq. We, therefore, used a Cas9 homology editing approach to generate endogenously tagged versions of our cell lines G523 and GSC3 by insertion of a FLAG tag at the N-terminal end of the protein (Fig. 6b). Precision of the edit was confirmed by Sanger sequencing and expression of the FLAG-tagged macroH2A2 from the endogenous locus was validated by immunofluorescence (Fig. 6a and Fig. S11a, b). Confocal microscopy confirmed nuclear localization of FLAG-tagged macroH2A2 (Fig. 6a). We performed ChIP-seq on G523 cells, and quality control measures showed a tight clustering of biological replicates with a distinct fingerprint plot, in keeping with a specific signal (Fig. 6c, d). We were able to identify both larger areas of signal and more focal peak regions (Fig. 6e). Motif enrichment analysis highlighted enrichment for mesenchymal-type transcription factors such as SNAIL1, SLUG, and ZEB2 (Fig. 6f), and gene ontology analysis suggested regulation of transcription factors, metabolism, and cellular differentiation and development (Fig. 6g). These results further support a role for macroH2A2 as a modulator of NPC/OPC and MES/AC cell states in GBM through regulation of chromatin programs.

We next set out to compare regions of macroH2A2 ChIP-seq enrichment with our ATAC-seq data, and found that there was a marked negative enrichment of ATAC-seq peaks in regions enriched for macroH2A2 (*p* < 0.001; hypergeometric test; Fig. 6h). In contrast, macroH2A2 peaks were enriched across a broad range of non-coding features including repeats, enhancers, and CTCF loop anchors (Fig. S11c–g). We performed an orthogonal analysis by looking for enrichment of macroH2A2-marked chromatin in regions that gained or lost accessibility upon macroH2A2 knockdown. This analysis showed that chromatin regions that lost accessibility were not enriched for macroH2A2-containing regions (Fig. S11h), but the peaks which gained accessibility showed some overlap (Fig. 6i). This suggests that the losses of accessibility seen upon knockdown are indirect and the effects of macroH2A2 itself on chromatin are mostly repressive. To further investigate these observations, we examined the overlap between our ATAC and macroH2A2 datasets in more detail, and found less overlap than expected by chance alone (*p* = 1.67e-56; Fisher exact test), with only 4167 overlapping peaks (Fig. 6j). Examination of relative distance also supported a relative negative enrichment of ATAC signal in regions marked by macroH2A2 (Fig. 6l).

We next set out to examine the effect of macroH2A2 on global chromatin organization, including areas outside of defined peaks. To do this, we examined the signals from both ChIP-seq and ATAC-seq experiments across the entire genome and correlated them genome-wide. This analysis revealed a higher correlation of chromatin accessibility and macroH2A2 ChIP signals in our knockdown cells compared to control cells (*p* = 0.006895), indicating slightly increased accessibility at macroH2A2-marked chromatin (Fig. 6k). As ATAC peaks gained upon macroH2A2 knockdown showed some overlap with macroH2A2, we further investigated ChIP-ATAC overlapping peaks, in order to identify which of these peaks were differentially accessible between control and *MACROH2A2* knockdown cells. An increased number of MACROH2A2-overlapping ATAC peaks were upregulated in our knockdown cells (Fig. S11g). Sorting these peaks by differential accessibility recovered a number of the regions we had noted earlier in our ATAC-seq data, including enhancers in the *COL20A1* and *FOXP1* genes (Fig. 6m), suggesting a direct role of macroH2A2 in repressing these regulatory regions. These results suggest that in GBM, the role of macroH2A2 at regulatory elements is primarily repressive, with knockdown resulting in a generalized increase in accessibility at macroH2A2-marked regions along with a subset of specific enhancer elements.

### Increasing macroH2A2 levels with a chemical compound reduces the stemness properties of GBM cells

**Fig. 7:**
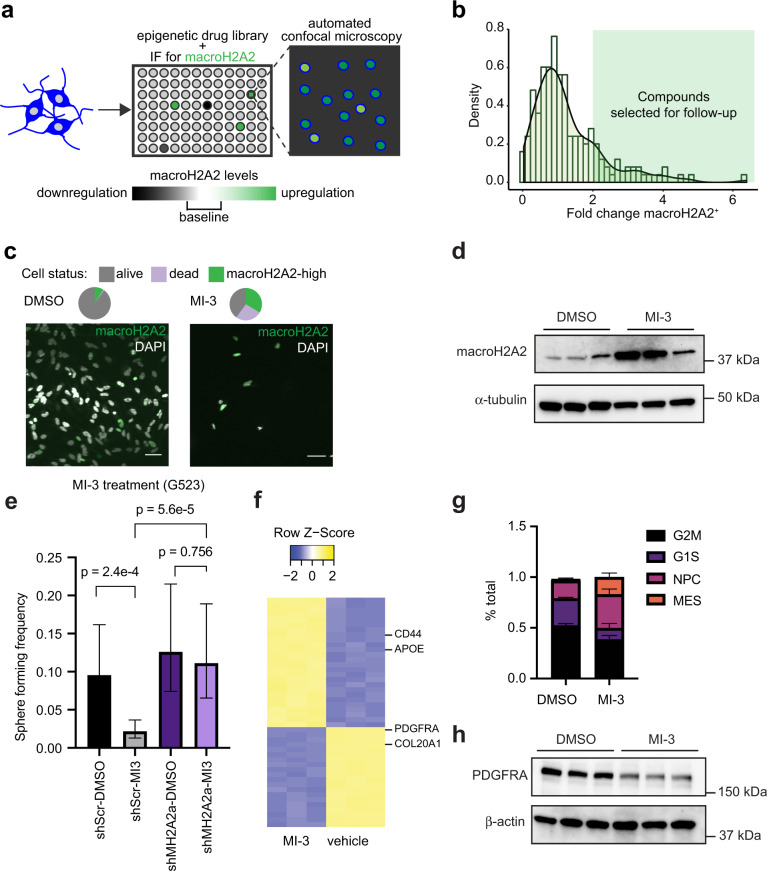
Identification of chemical compounds that elevate macroH2A2 levels in GBM cells. **a** Diagram summarizing our screening strategy to identify compounds that increase macroH2A2 levels. **b** Normalized density of the log fold change of macroH2A2 positive cells for all compounds in the screen. The green-shaded region represents compounds with greater than a twofold change of macroH2A2 positive cells. **c** Effects of MI-3 at 1 uM and vehicle control (dmso) on macroH2A2 protein levels were assessed by immunofluorescence. Scale bars: 50 mm. Representative images from one of three biological replicates. The experiment was performed once. **d** Western blot of macroH2A2 levels after 7 days of treatment with 200 nM of MI-3. Three replicates per condition. **e** Limiting dilution assay of macroH2A2 knockdown cells versus control GSCs treated with either DMSO or 500 nM of MI-3. Sphere formation estimates from *n* = 6 biological replicates. *P* value determined by Chi-square test. Error bars represent a 95% confidence interval of the sphere formation frequency estimate. **f** Heatmap displaying the top 50 differentially expressed genes between MI-3 and DMSO-treated cells based on RNA-seq data (three biological replicates per treatment). Error bars represent standard deviation. **g** CIBERSORTx analysis of transcriptional subtypes in MI-3 data (*p* values: G2M 0.0129; G1S 0.024; NPC 0.029; MES 0.022; two-tailed unpaired *T*-test with Welch’s correction; *n* = 2 biological replicates per condition). **h** Western blot showing levels of PDGFRA and TNFR in MI-3 and DMSO-treated GBM cells after 7 days of treatment in vitro. Three biological replicates per condition. The experiment was repeated two times.

As we noticed that macroH2A2 appeared to repress self-renewal programs in GBM, we reasoned that increasing macroH2A2 could lead to the “differentiation” of GBM cells. We, therefore, set out to perform a chemical screen with the goal of identifying compounds that could increase levels of macroH2A2 (Fig. 7a and Fig. S12a). We screened 182 compounds from the Selleckchem Epigenetic Drug Library using the GE InCell automated confocal microscopy system, coupled with immunofluorescence to assess macroH2A2 protein levels in individual cells (Fig. 7a). We were able to identify 35 compounds that led to greater than twofold increase in the percentage of macroH2A2^+^ GBM cells. These included MI-3 (an inhibitor of interactions between MLL1 and menin)^87,88^ and RGFP-966 (an HDAC3-selective histone deacetylase inhibitor) (Fig. 7b, c and S12b)^89^. In follow-up experiments, we confirmed that both compounds had potent effects on cell viability (Fig. S12c, d), supporting the robustness of our screen. We validated that MI-3 increases macroH2A2 levels by western blot (Fig. 7d). Moreover, treatment of GSCs with MI-3 reduced sphere formation in LDA experiments, an effect that was markedly abrogated by the knockdown of *MACROH2A2* (Fig. 7e). Given the ability of MI-3 to increase macroH2A2 protein levels and repress self-renewal in a macroH2A2-dependent fashion, we decided to use this chemical compound to further interrogate the molecular function of macroH2A2 in GBM.

In the first set of experiments, we pursued a transcriptomics strategy to unravel transcriptional programs downstream of chemically-induced increased expression of macroH2A2. We treated GBM cells with sub-lethal concentrations (200 nM) of MI-3 or DMSO vehicle control and performed transcriptional studies by RNA-seq (three biological replicates per condition). Our transcriptomic experiment showed that MI-3 treatment results in the repression of markers of the oligodendrocytic lineage, including *PDFGRA* and *COL20A1*, and increased expression of markers of the astroglial lineage, such as *CD44* and *APOE* (Fig. 7f). We performed decomposition analysis to further interrogate the effects of MI-3 on cell states (Fig. 7g), and found that there was a marked reduction in cycling cells in G1S (*p* = 0.0129) and G2M (*p* = 0.0237) with an increase in MES (from 2 to 17%; *p* = 0.02245) and increase in NPC-associated cells (17.5 to 33.3%; *p* = 0.0293). We suspect the increase in NPC-type cells is likely at least partly a result of the reduced numbers of cycling cells, and may reflect cells which have exited the cell cycle. Western blot and RT-qPCR also confirmed the downregulation of PDGFRA following MI-3 treatment (Fig. 7h and Fig. S13c). These results are the opposite of what we saw upon *MACROH2A2* shRNA-induced knockdown (Fig. 2b), and further confirm the robustness of our strategy and that the activity of MI-3 is at least partially mediated by macroH2A2.

**Fig. 8:**
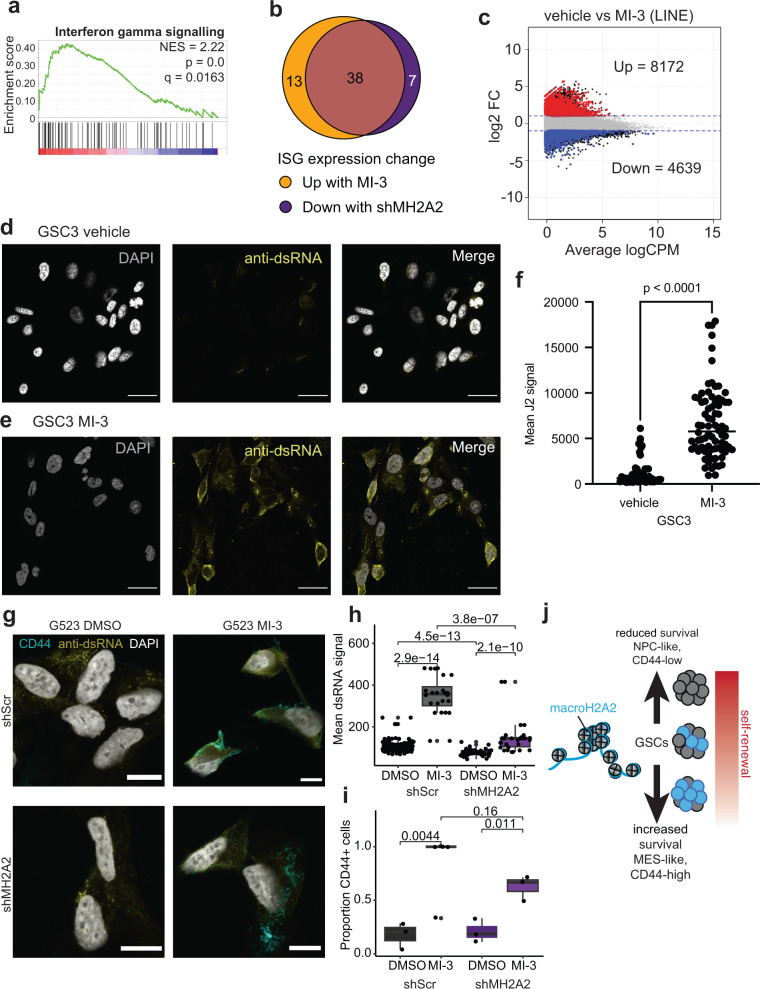
macroH2A2 is a positive modulator of viral mimicry pathways in GBM. **a** GSEA analysis showing increased interferon signaling in MI-3-treated versus vehicle-treated cells. *P* value represents the hypergeometric test and *q* value represents the false-discovery corrected *P* value. **b** Overlap of interferon-sensitive genes (ISGs) showing expression changes upon MI-3 treatment compared to ISGs differentially expressed upon macroH2A2 knockdown. **c** Transcriptional levels of LINE repeat elements upon MI-3 treatment were determined by RNA-seq. **d**–**f** Staining for double-stranded RNA in vehicle-treated (**g**) and MI-3 treated (**h**) GSC3 GBM cells. Scale bar: 25 mm. **i** Quantification of dsRNA signal per cell in vehicle versus MI-3 treated cells. *P* value obtained by Mann–Whitney test. The experiment was repeated twice in two different cell lines (G523, GSC3). **g–i** Immunofluorescence staining for double-stranded RNA and CD44 (**g**) in vehicle treated versus MI-3 treated control or shMH2A2 G523 GBM cells. Scale bar: 10 microns. **h** Quantification of dsRNA signal in vehicle versus MI-3 treated cells per cell in at least *n* = 3 60x fields per condition. *P* value obtained by two-tailed two-tailed unpaired *T*-test with Welch’s correction. The boxplot line represents the median, hinges at 25th and 75th percentiles, and whiskers at 1.5 × IQR. **i** Quantification of the proportion of CD44-positive cells in at least *n* = 3 60x fields in each condition. The boxplot line represents the median, hinges at 25th and 75th percentiles, and whiskers at 1.5 × IQR. *P* value obtained by two-tailed two-tailed unpaired *T*-test with Welch’s correction. The experiment was repeated twice. **j** Proposed model for the mechanisms of action of macroH2A2 in GSCs.

We performed GSEA and found that differentially-regulated genes following MI-3 treatment were positively associated with signatures of interferon-gamma, alpha and beta signaling (Fig. 8a and Fig. S13a) and negatively associated with signatures linked to PRC2-mediated methylation of histones (Fig. S13b). Modulation of interferon signaling related to altered DNA methylation has been reported with epigenetic treatments that elicit viral mimicry in cancer cells and is a hallmark of the mesenchymal state^89,90^. Viral mimicry is dependent on the transcription of repetitive elements across the genome, particularly ERVL elements^91^. We, therefore, re-analyzed our RNA-seq datasets to specifically look at the effects of MI-3 treatment on the transcription of repetitive elements. We found that most differentially transcribed repetitive elements were upregulated by MI-3 treatment, including LINE elements and ERVL family members (Fig. 8c and Fig. S13f). This was associated with a concomitant increase in the expression of interferon-sensitive genes (ISGs) (Fig. 8b and Fig. S13e). In contrast, when we examined the expression of the same set of ISGs in macroH2A2 knockdown cells, we found a majority of the same genes (38 of 45 genes) were downregulated in macroH2A2 knockdown GSCs (Fig. 8b and Fig. S13d). Our orthogonal methods (a chemically-induced increase of macroH2A2 levels and shRNA-mediated downregulation of this histone variant) indicate that macroH2A2 is associated with a state of viral mimicry in GBM cells.

In a second set of experiments, we further tested the link between MI-3 treatment and viral mimicry. Induction of viral mimicry is accompanied by increased expression of double-stranded RNA (dsRNA). Staining for dsRNA in control versus MI-3-treated cells found markedly greater staining for dsRNA in treated versus control cells (Fig. 8d–f; *p* < 0.0001 by Mann–Whitney test**)**. To further investigate the interaction between the effect of macroH2A2 knockdown and the dsRNA response, we repeated this experiment in knockdown and control cells, alongside CD44 labeling. This showed a significant reduction of dsRNA levels at baseline in shMH2A2 knockdown cells (*p* = 4.5e-13), and a significant increase upon MI-3 treatment in both conditions (*p* = 2.9e-14; *p* = 2.1e-10; two-tailed unpaired *T*-test), albeit with a markedly attenuated response in the mH2A2-knockdown cells (*p* = 3.6e-7 for MI-3 shMH2A2 versus shScr). This shift was accompanied by an increase in CD44-positive cells, which was more prominent in the shScr condition (*p* = 0.0044 for shScr; *p* = 0.011 for shMH2A2). These data suggest that macroH2A2-mediated state shifts modulate the cell’s capacity to produce and respond to dsRNA. Given that overexpression of macroH2A2 did not result in reduced viability in our models, this suggests that macroH2A2 expression does not directly upregulate dsRNA molecules, but more likely associates with cell states with greater vulnerability to stress-mediated dsRNA production. Overall, our data indicate that it is possible to increase macroH2A2 levels using chemical compounds, which can induce differentiation and cell death of GBM cells (Fig. 8j).

## Discussion

Self-renewal is a fundamental property of cancer cells that are therapy-resistant and responsible for long-term tumor propagation. The identification of molecular mechanisms that are responsible for the attainment and maintenance of self-renewal could be key to significantly improve patient outcomes for difficult-to-treat cancers like GBM^92^. Self-renewal in GBM cells is dependent on chromatin states and transcriptional profiles achieved through the regulated function of chromatin remodelers, transcription factors, and other epigenetic regulators^10,23,31,32,34,93^. Interfering with chromatin and epigenetic factors that have key roles in self-renewal programs could destabilize these chromatin states, abrogate stem-like cancer cells, and, consequently, improve therapy response.

In the present work, we identify macroH2A2 as a potent antagonist of self-renewal properties in GBM. This conclusion is supported by multiple orthogonal lines of evidence. First, high *MACROH2A2* transcript levels are associated with better response to therapy and longer overall survival in GBM patient cohorts. The prognostic value of macroH2A2 expression levels is particularly strong in the proneural molecular subtype, which represents about a third of GBM patients^59^. These effects of *MACROH2A2* were recapitulated in our in vivo orthotopic patient-derived models, showing direct functional involvement of this gene in regulating tumor aggressiveness. Second, our in vitro and in vivo functional assays demonstrated that macroH2A2 has a direct functional role in curbing self-renewal. Our genomic approaches showcase the function of macroH2A2 in shaping chromatin organization at enhancer elements and regulating the expression of a self-renewal gene network. Overall these results indicate that macroH2A2 has an important effect on the regulation of chromatin and transcriptional dynamics that oppose self-renewal in GBM.

Previous reports noted that macroH2A2 acts as a barrier to reprogramming of somatic cells to induced pluripotent cell states^55,94^. macroH2A2 might therefore have a conserved role in antagonizing self-renewal properties in neoplastic and non-neoplastic cells. Here, we provide evidence supporting such a role for macroH2A2 in GBM. A function of this histone variant in antagonizing stemness programs may be applicable to other cancer paradigms. For instance, macroH2A2 expression tends to be repressed in melanoma. Loss of macroH2A2 expression in melanoma cells was associated with higher levels of CDK8, an established oncogene^36^. It remains to be determined whether macroH2A2 antagonizes stemness programs in melanoma, as it does in GBM.

How macroH2A2 antagonizes transcriptional programs of stemness is likely complex, with both direct and indirect effects on gene expression. We combined data from ATAC-seq and macroH2A2 ChIP-seq experiments to further interrogate this relationship, and our data suggest that macroH2A2 largely shapes chromatin accessibility to modulate the function of enhancer elements. We propose that macroH2A2 antagonizes self-renewal at least partially through the repression of cis-regulatory regions associated with stemness programs. The effects of macroH2A2 on chromatin accessibility are heterogeneous, with areas of both gained and lost accessibility genome-wide. However, our integration of macroH2A2 ChIP-seq data and ATAC-seq data suggests that this histone variant is primarily involved in repressing chromatin. The apparently paradoxical role of this protein in also maintaining accessible chromatin—as we and others^36^ have reported—is likely an indirect effect dependent on other contextual chromatin networks.

Our functional assays support the notion that macroH2A2 orchestrates a chromatin shift that represses stemness programs in GBM. Our experimental data are consistent with the association between high macroH2A2 transcriptional levels and better prognosis we observed in GBM clinical cohorts. These tumors might have a greater proportion of differentiated cells and fewer stem-like/self-renewing cells, a tumor composition that would also explain why macroH2A2-high tumors respond better to therapy.

We report that *MACROH2A2* expression is enriched in NPC-like cells compared to AC and MES states. This seems in conflict with the observation that knocking down macroH2A2 results in an increased fraction of cells transitioning to NPC/OPC states. We think that these counterintuitive results could be reconciled by our observation that macroH2A2 is expressed in a subset of NPC-like cells that are actively cycling. Reduction of macroH2A2 levels pushes cells to exit the cell cycle—based on our in vivo data – a phenotype that is consistent with more primitive stem-like states in GBM^11,95,96^. This scenario would explain why the knockdown of this histone variant results in increased self-renewal, as assessed in vitro and in vivo. It is also consistent with a recent publication that showed NPC/OPC-like cells playing a major role in the invasion of non-neoplastic tissue^97^, a behavior previously demonstrated in stem-like cells^98^, whereas MES-like cells localize to the tumor core. It appears that macroH2A2 is predominantly expressed in NPC/OPC-like cells, and its modulation can shift cells to either a more extreme NPC/OPC phenotype, by reducing macroH2A2 levels, or toward an AC/MES phenotype, with increasing macroH2A2 levels. In this way, modulating macroH2A2 levels in the context of the NPC-like state can therefore transition cells along a gradient of self-renewal/stemness.

A smaller fraction of self-renewing, therapy-resistant tumor cells could at least partially explain the better prognoses of macroH2A2-high tumors. A second factor that could explain their better prognosis could be associated with an increased ability to activate viral mimicry programs. Our data demonstrated that treatment with a small molecule elevates macroH2A2 levels and robustly activates viral mimicry responses in GBM cells, and the effect of this compound is largely abrogated by macroH2A2 knockdown. Given the marked effect of knockdown on the fraction of CD44-positive cells and the significant increase of CD44 seen upon MI-3 treatment, we suspect that GBM sensitivity to MI-3 may be state-specific. Activation of macroH2A2 may enable transitions of cells into states that are sensitive to an MI-3-induced viral mimicry response. Viral mimicry was previously associated with the expression of dsRNA following the administration of DNA demethylating agents^89^. Orthogonal evidence supports this notion, including (i) increased expression of ISG genes following increased macroH2A2 levels, (ii) decreased expression of ISG genes upon macroH2A2 knockdown, and (iii) increased production of dsRNA from repetitive sequences upon MI-3 treatment. Increased CD44 and ISG expression are also features of the MES states, and it is unclear to what extent the viral mimicry response we observe is a byproduct of increased MES-type cells, a direct drug-induced viral mimicry response, or a combination of both factors. Increased intracellular dsRNA with an activated, CD44-high astrocytic phenotype has been noted in non-neoplastic astrocytes in certain neurodegenerative conditions, such as TDP43 knockdown models of amyotrophic lateral sclerosis^99^. This suggests that dsRNA production may be an intrinsic property associated with an intracellular stress response in some GBM cells. Our results show that activation of the macroH2A2 histone variant is associated with cell states capable of viral mimicry responses. While these changes are associated with increases in macroH2A2 levels, they may not be a direct consequence of macroH2A2 expression, but rather a reflection of an underlying state transition.

In conclusion, our work characterizes an epigenetic mechanism of self-renewal regulated by the histone variant macroH2A2. Given that the histone variant H3.3 was previously shown to also repress stemness in GBM^10^, we postulate that a histone variant code might contribute to chromatin programs that modulate the stemness properties of cancer cells. Our data underscore the close connection between histone variants and chromatin and functional states in cancer cells.

## Methods

### Ethics approval

All animal studies were performed at the University of Calgary and approved by the University of Calgary Animal Care Committee (study identification number AC21-0226). The acquisition and use of tumor samples was approved by the Health Research and Ethics Board of Alberta (HREBA) (study identification number HREBA.CC-16-0823).

### Experimental model and subject details

#### Primary glioma patient cell cultures

All specimens and primary cultures generated and used in this study were approved by the Health Research Ethics Board of Alberta and the research ethics board of the Hospital for Sick Children (Toronto, ON). GSC primary cultures G523, GSC2, and GSC3 were generated using previously described methods; in brief, the primary tumor sample was minced in Accutase (StemCell Technologies), and dissociated with glass beads on a nutator for 30 min, followed by centrifugation and resuspension in NS media^25^. Cultures were STR genotyped and confirmed to match the patient tissue. Mycoplasma testing was performed using the Lonza Mycoalert Mycoplasma Detection Kit (LT07-318) and all lines used for studies were mycoplasma-negative. Additional specimen information is available in Supplementary Table 1. Cell cultures can be made available upon reasonable request.

#### Sex and gender considerations

Information on patient sex was collected as part of the tumor banking process, and gender was not considered in our analyses. Sex-based analysis was not performed as analysis of cohorts showed no differences in macroH2A2 expression between male and female patients.

### Cell culture

Primary glioblastoma cultures were grown in adherent culture on Corning Primaria dishes coated with poly-L-ornithine (Sigma-Aldrich, P4957) and laminin (Sigma-Aldrich, L2020) under standard temperature, oxygen, and humidity conditions. Cells were kept in NeuroCult NS-A Basal Medium and Proliferation Supplement (StemCell Technologies, #05751), supplemented with 20 μg/mL rhEGF (Peprotech, AF-100-15), 10 μg/mL bFGF (StemCell Technologies, #78003), and 2 μg/mL heparin (StemCell Technologies, #07980). All cultures were used within the first 20 passages of generation. Adherent cells were disassociated with Accutase (StemCell Technologies, #07920) and plated onto fresh, coated plates when confluence reached 80/90%. Cell numbers and viability were determined using Countess II (Thermo Fisher Scientific, AMQAX1000).

### Generation of macroH2A2 knockdown cultures

Commercial inducible shRNA constructs (3 for *MACROH2A2*, and non-targeting control 1) were obtained from Dharmacon (Horizon Biosciences) and packaged into lentiviral particles. Primary cells were infected with lentiviral particles in the presence of polybrene, followed by selection using 1.5 μg/mL puromycin for 72 h.

### MacroH2A2 overexpression with CRISPRa

#### Generation of an inducible overexpression line

The PB-TRE-dCas9-VPR construct (Addgene #63800)^73^ was used to create a stable line using Piggybac transposase (System Biosciences). A total of 500,000 cells were transfected using the mouse neural stem cell nucleofection kit (Lonza, VPG-1004) with 0.66 μg of the construct and 0.33 μg of transposase using the Lonza Amaxa Nucleofector I with protocol A-33, followed by selection with hygromycin B at 50 ug/mL for 7 days.

#### Generation of sgRNA plasmids

Guide RNAs were designed using the crispr.mit.edu guide design tool, using as templates the DNAse accessible region directly upstream of the TSS of *MACROH2A2*. The plasmid backbone pLKO-sgRNA-GFP (Addgene #57822) was used and sgRNA plasmids were constructed using BsmBI digestion followed by ligation, as previously described in ref. ^100^.

#### Transfection for overexpression experiments

Two million cells containing the dCas9-VPR construct were transfected with a pool of multiple guides targeting *MACROH2A2* or a non-targeting control using the Amaxa Nucleofector. Doxycycline (2  μg/mL) was added to the media 24 h after transfection.

### In vitro limiting dilution analysis

Cells were plated on uncoated low-adhesion 96-well plates in a twofold dilution series spanning from 2000 down to four cells per well in NeuroCult NS-A media (StemCell Technologies, #05751) containing doxycycline at 2 μg/mL, with six replicates per concentration. Sphere formation frequency was estimated using ELDA^101^. Sphere formation was scored on day 7 and day 14.

### RT-qPCR

RNA samples were used to generate cDNA using the SuperScript II kit (Invitrogen) and poly-A primers. PCR was performed using the SSOFast EvaGreen Supermix (Bio-Rad # 1725201) on the Bio-Rad CFX with all samples in triplicate. Results were analysed using the delta Ct method.

### Western blot

The protein concentration of samples was determined using the DC (detergent compatible) protein assay (Bio-Rad, #5000112). Samples were prepared in a total volume of 20 μL at 15 μg/μL in Laemmli loading buffer. Samples were run on 7.5% Mini-PROTEAN gels for cytoplasmic proteins and 12.5% Mini-PROTEAN gels for histones (Bio-Rad, #456025). Primary antibodies used: Rabbit anti-mH2A2 (Invitrogen, PA5-57437) at 1:250 dilution, mouse monoclonal anti-β-ACTIN (Sigma-Aldrich, A5441, lot # 127M4866V, clone AC-15) at 1:1000, rabbit anti-lamin A/C (Abcam, ab108595) at 1:1000, rabbit anti-OLIG2 (Millipore, AB9610) at 1:500, mouse anti-GFAP (Millipore, MAB360), rabbit anti-H3 (Cell Signaling Technology, #9715) at 1:500, rabbit anti-PDFGRA (Cell Signaling Technology, #3164) at 1:500, rabbit polyclonal anti-macroH2A1 (Millipore, ABE215) at 1:500. Secondary antibodies used: Goat anti-rabbit IgG H&L (HRP) (Abcam, #6721, lot # GR3192725-6) at 1:20,000 dilution, goat anti-mouse IgG H&L (HRP) (Abcam, #6789) at 1:2000 dilution.

### Mouse intracranial orthotopic xenografts

#### Mouse husbandry

Female NSG mice (Jackson stock no 005557) were grown in a clean facility with temperature maintained between 22–25 °C and humidity of 30–70%, with light-dark cycles of 12/12 light/dark with light exposure occurring from 7 a.m. to 7 p.m. daily.

#### Mouse survival

For each mouse, 100,000 tumor cells (control or knockdown) in PBS were stereotactically injected into the forebrain (Location: 2.0–3.0 mm to the right of bregma, 1.0 mm anterior to coronal suture) of 3-month-old female NSG mice (Jackson Stock no 005557), using a 30 gauge needle. Mice were fed 2 mg/mL doxycycline in a 2% sucrose water solution. Endpoint was reached once mice showed signs of disease, including ataxia, hunching, domed heads, kyphosis, paresis and lethargy, poor oral intake, or weight loss of greater than 15%. Mouse tumors remained intracranial and did not exceed the tumor burden limits determined by the institutional review board.

#### Orthotopic limiting dilution assay

Stereotactic injections of 100,000, 10,000, or 1000 cells were performed into the right forebrain of NSG mice as described above. Mice were sacrificed at the endpoint as in the previous experiment.

All animal work was approved by the Animal Care Committee at the University of Calgary.

### Immunofluorescence microscopy

#### Immunohistochemistry of primary patient samples

Slides were cut to a thickness of 4 µ and dried in a 56 °C oven overnight. Samples were deparaffinized with xylene and dehydrated, followed by peroxidase treatment for 15 min (3% H_2_O_2_), and antigen retrieval with EDTA buffer (1 mM EDTA pH 8.0; 0.05% Tween-20) in a pressure cooker for 35 min. Samples were blocked with BSA (5% BSA in PBS with 0.1% Triton X-100) for 1 h, then incubated with primary antibody (rabbit anti-mH2A2: 1:250, Novus NBP1-92094; rabbit anti-mH2A1 1:1000, Millipore ABE215) overnight at 4 °C in staining buffer (5% BSA in PBS and 0.1% Tween-20), followed by incubation with a secondary antibody (goat anti-rabbit HRP; Abcam ab6721) for 30 min at room temperature. Washes were performed using PBS with 0.1% Tween. DAB staining was applied for 3 min (Dako/Agilent K346811-2), followed by washing and hematoxylin counterstaining.

#### Immunofluorescence of primary patient samples

Initial processing was done as above, but without peroxidase treatment. Samples were blocked with BSA (5% BSA in PBS with 0.1% Triton X-100) for 1 h, then incubated with primary antibody (Rabbit anti-mH2A2: 1:250, Novus NBP1-92094; Mouse anti-SOX2: 1:500, Cell Signalling Technologies #2748) overnight at 4 °C in staining buffer. A secondary antibody was applied the next day (Invitrogen Goat anti-mouse Alexa Fluor 568 [A11011] and Donkey anti-rabbit Alexa Fluor 647 [A31573]) at 1:500 for 1 h in the dark. Sections were then incubated in DAPI (dilution 1:1000; Thermo Fisher #62248), washed in PBS, and mounted on 1.5 glass coverslips with Prolong Diamond antifade (Life Technologies P36965). Images were acquired on the ZEISS LSM 880 Airyscan confocal microscopy (Zeiss).

#### Xenograft microscopy

Xenografted brains were fixed at an endpoint in 4% PFA overnight, followed by overnight treatment in 30% sucrose in PBS, after which they were embedded in molds with Sakura TissueTek OCT (Thermo Fisher 14-373-65). Sections were stained overnight in 5% BSA in PBS with 0.01% Tween at 4 °C, followed by incubation with a secondary antibody (Invitrogen anti-rabbit Alexa A488), and anti-CD44 APC-conjugated antibody (Miltenyi Biotec 130-113-338; dilution 1:50) for the CD44 experiments, for 1 h at room temperature. Antibodies used were as follows: mouse anti-human nuclei antibody (Millipore MAB1281; dilution 1:200), rabbit anti-Ki-67 (Abcam ab16667, dilution 1:200), mouse anti-Ki-67 (BD #550609), rabbit anti-ASCL1 (Cell Signalling Technologies #55467; dilution 1:100), rabbit anti-OLIG2 (Millipore AB9610; dilution 1:500), and rabbit anti-macroH2A2 (dilution 1:250, Novus NBP1-92094). Washes were performed using 0.01% Tween. Sections were incubated with propidium iodide (dilution 1:1000; Thermo Scientific; P3566) in PBS for 10 min or DAPI (dilution 1:1000; Thermo Fisher #62248) for 5 min, followed by a PBS wash. Slides were mounted using FluorSave reagent (Fisher 345789). Images were acquired on the EVOS FL Auto (Thermo Fisher) for wide-field imaging or on the ZEISS LSM 880 Airyscan confocal microscopy (Zeiss).

#### Xenograft image analysis

Image analysis was performed using Fiji and R. For nuclear markers, initial masking of images was performed based on a dual thresholding method using a binary combination of the tool Find Maxima to output segmented particles between cell maxima, and Threshold using Otsu’s method, followed by the Analyze particles tool to identify segmented nuclei. Measurements of all channels were acquired for at least three 10x fields on a wide-field microscope (Ki-67; SOX10; OLIG2) or 20x confocal regions (macroH2A2) for each condition. Additional analyses were performed in R, and thresholding of cells as positive or negative was done by application of Mclust to automatically determine a threshold for positive versus negative cells across the samples. For membranous markers (CD44), quantification was performed by applying the Threshold tool using Otsu’s method, and then applying the Measure tool to identify the % of the area of each 10x field, which was positive. Statistical differences were tested using two-tailed unpaired T-tests and Chi-squared tests for proportions between control and knockdown samples.

#### Immunocytochemistry

Cells were grown on 12 mm German glass coverslips (VWR 89167-106) coated with poly-l-ornithine (Sigma-Aldrich, P4957) and laminin (Sigma-Aldrich, L2020), and treated with either 200 nM MI-3 or DMSO for 7 days. Cells were fixed in 4% PFA in PBS for 10 min at room temperature. Coverslips were blocked in 5% BSA in PBS with 0.1% Tween for 1 h at room temperature. Staining was performed overnight at 4 °C in the same media with the mouse anti-J2 (anti-dsRNA) antibody (Scicons) at a dilution of 1:200, followed by labeling with a fluorescently conjugated secondary antibody (Invitrogen Goat anti-mouse IgG A568, A11011), and a DAPI counterstain (1:1000; Thermo Fisher #62248). Washes were performed using PBS with 0.1% Tween. Staining for FLAG was performed using an anti-FLAG antibody (CST CST #14793) at 1:500 overnight with donkey anti-rabbit Alexa Fluor 647 [A31573]) at 1:500 for 1 h as the secondary antibody. Images were acquired using the ZEISS LSM 880 Airyscan confocal microscope (Zeiss).

#### J2 image analysis

Images were thresholded in Fiji to identify individual cells, and mean and total fluorescence signal were analysed. Statistical analysis between the MI-3 and DMSO-treated groups was performed in GraphPad Prism, using the Mann–Whitney test for significance.

### Analysis of published datasets

#### Survival analysis

Data from The Cancer Genome Atlas GBM samples and the Gravendeel et al. dataset were downloaded from R2. Samples were thresholded by the median expression value of the gene of interest (e.g., *MACROH2A2* or other histone variant genes), and survival analysis was performed in R using the tool survminer. Cox proportional hazards analysis was performed using the coxph function in survminer, and plots were generated using ggforest and ggadjustcurves.

#### Analysis of published RNA-seq, scRNA-seq, and scATAC-seq datasets

Data from ref. ^8^ and ref. ^16^ was downloaded from the Broad single-cell portal and analysed in R. For G523 cell states, data were obtained from the AUCs in the Richards et al. metadata. For GSE139261^90^, data were downloaded from GEO and analyzed in R to examine levels of macroH2A2 in different groups. For GSE117891^68^, data matrices were obtained from GEO, followed by calling of inferCNV^102^ to separate neoplastic and non-neoplastic cells, followed by plotting of normalized counts for *MACROH2A2* in tumor cells from peripheral or central samples. For single-cell ATAC-seq data (GSE139136)^78^, subtypes were inferred by applying CellAssign^103^ to GeneActivity scores generated using Signac^104^. Peaks were subset by those which were also present in our ATAC-seq consensus peak set and used to generate a single-cell reference matrix for CIBERSORTx.

### ATAC-seq

#### Experimental method and sequencing

ATAC-seq was performed using the Omni-ATAC protocol^77^. In brief, 50,000 cells were harvested fresh from culture from two biological replicates, lysed on ice, spun at 4 °C, and treated with Tn5 transposase (Illumina) for 30 min at 37 °C. Libraries were amplified using the NebNext HiFi polymerase mastermix (New England Biolabs) and standard Illumina Nextera primers, and DNA was purified using the MinElute kit (Qiagen). Sequencing was performed on a NextSeq 500 using 150 cycles of paired-end 75 bp sequencing at the Center for Health Genomics and Informatics (University of Calgary).

#### ATAC-seq data analysis

ATAC-seq data were aligned to hg38 using bwa 0.7.17 with the mem algorithm^105^. Following this, samtools was used to remove the mitochondrial and Y chromosome reads and reads mapping to genome blacklists^106^. Duplicates were removed using Picard. Peak assignments were generated using macs2^107^. Differential peak calls were performed by generating a pileup of counts of all consensus peaks from all samples, transforming the counts to counts per million using edgeR, and running these normalized counts through DESeq2^108^. Peaks with a fold change of >1.5 were considered significantly altered. Changepoint analysis was run on a pileup of ATAC signal reads binned into 1 mb bins using bedtools, using the R package *changepoint*, as previously described in ref. ^10^. Permutation analysis was performed using the R package regioneR^109^. Gene ontology analysis was performed using DAVID 6.8^110,111^ and motif enrichment was calculated using HOMER^79^. CIBERSORTx^112^ was used to infer subtypes, using a custom single-cell ATAC-based reference.

### RNA extraction and RNA sequencing

#### Sample preparation and sequencing

For macroH2A2 knockdown RNA-seq, stably transduced cells were induced with 2 μg/mL doxycycline and grown for 7 days in culture and processed in biological duplicates. For MI-3 vs DMSO-treated cells, G523 cells were treated with either 200 nM MI-3 or DMSO for 7 days and processed in biological triplicate. Cells were harvested using Accutase, and RNA was extracted using the RNEasy Mini kit (Qiagen) as per manufacturer instructions. Libraries were constructed at the Center for Health Genomics and Informatics using the NEBNext Ultra II Directional RNA Library prep kit (New England Biolabs) with ribosomal RNA depletion. Samples were sequenced on a NextSeq 500 for 150 cycles in a single-end mode for mH2A2 knockdown, and paired-end mode for MI-3.

#### RNA-seq analysis

Samples were pseudoaligned to the human transcriptome (GRCh38.rel79) using Kallisto, and differential analysis was performed using sleuth^113,114^. GSEA was performed using a ranked list of all genes generated using sleuth. For analysis of eRNAs, the Fantom5 CAGE^115^ consensus list of enhancers, as well as a custom list of predicted enhancers based on G523 ATAC-seq and H3K27ac consensus peaks, was used to construct a custom pseudotranscriptome, which was analysed using kallisto and sleuth in a similar fashion. Analysis of repeat expression was performed using REdiscoverTE^116^. CIBERSORTx^112^ was used to perform the decomposition of cell states using a single-cell reference matrix generated using data from Neftel et al. As the algorithm was unable to distinguish between NPC1/NPC2 and MES1/MES2 in our results, we have designated these NPC and MES, as these may represent either of these cell types. Heatmaps were constructed with R using the heatmap.2 function from the package gplots.

### ChIP-seq sample preparation and sequencing

#### Generation of stable endogenously tagged macroH2A2 lines

Guide RNAs targeting the N-terminal end of human macroH2A2 and homology arms were designed using CHOPCHOP^117^, and the homology template was modified by the addition of a 2xGGS linker and a FLAG tag. Custom crRNAs were ordered and transfected into 500,000 cells using the Alt-R CRISPR-Cas9 system (Integrated DNA Technologies). Two days after transfection, cells were plated in serial dilution on a PLO-laminin-coated 96-well plate (Falcon), and allowed to grow. Single clones were isolated, validated by Sanger sequencing and immunocytochemistry, and expanded.

#### Chromatin immunoprecipitation

Cells were grown under standard culture conditions in 10 cm PLO-laminin-coated Primaria plates. Immunoprecipitation was performed based on published protocols^10^, with some modifications. In brief, cells were resuspended in warm media, then fixed in fresh PFA to a final concentration of 0.5% for 5 min with agitation, followed by quenching with 750 mM Tris-HCl pH 8.1. Nuclei were then extracted on ice with nuclear extraction buffer (10 mM Tris-HCl pH 7.5; 10 mM NaCl; 0.3% Igepal CA630; 3 mM MgCl_2_, Roche protease inhibitors), and cells were spun to isolate nuclei. Nuclei were lysed in lysis buffer (1% SDS; 10 mM EDTA; 50 mM Tris-HCl pH 8.1), then sheared on a Diagenode Bioruptor for 40 min at high power and 30 s on, 30 s off. Immunoprecipitation was performed overnight at 4 °C on an equivalent of 1–2 million cells, with 20 uL of protein A Dynabeads (Invitrogen) with 8  uL of anti-FLAG antibody (Cell Signalling Technologies #14793). Samples were washed on a rotator at 4 °C for 5 min each with low-salt (20 mM Tris-HCl pH 8.0, 2 mM EDTA; 150 mM NaCl, 1% Tx100, 0.1% SDS), high-salt (20 mM Tris-HCl pH 8.0, 2 mM EDTA; 500 mM NaCl, 1% Tx100, 0.1% SDS), 250 mM RIPA-LiCl (50 mM HEPES pH 7.4, 1 mM EDTA; 0.7% Na deoxycholate, 1% NP-40, 250 mM LiCl) and TE buffers supplemented with protease inhibitors (Roche). Samples were eluted in elution buffer (1% SDS; 10 mM Tris-HCl pH 8.0, 5 mM EDTA pH 8, 300 mM NaCl) with RNAse (20 μg/mL) and Proteinase K (20 μg/mL) in a dry bath for 6 h at 65 °C with agitation. DNA was purified using SPRI beads (Beckman Coulter) with a 1.8X ratio and eluted in 10 mM Tris-HCl pH 8.0.

#### Library construction and sequencing

Libraries were constructed at the Center for Genomics and Health Informatics (CHGI) using the NEBNext Ultra II DNA Library Prep Kit (New England Biolabs). Sequencing was performed at the CHGI using an Illumina NovaSeq 6000 on an SP flow cell with 200 cycles (2 × 100 bp).

#### ChIP data analysis

Samples were aligned using bwa 0.7.17^105^ with the mem algorithm to the GRCh38 (hg38) human reference genome. Additional filtering was applied using samtools^106^ to remove reads mapping to blacklist regions and reads with *q* < 30. Duplicates were marked and removed using Picard. Peaks were called using sicer^118^ with default parameters and using an input sample as a reference. Peaks from different replicates were merged to create a consensus peaklist using bedtools merge^119^. Additional quality control was performed using deeptools 2.2.0^120^, using the tools multiBamSummary, and plotCorrelation (using a spearman correlation), as well as plotFingerprint. Pileups for visualization were generated using the deeptools command bamCompare, using the settings -bs 20, -smoothLength 100–operation ratio. Permutation testing was performed using regioneR, as for ATAC-seq. Motif calling and gene ontology analysis was performed using HOMER^79^.

#### Comparison of ATAC and ChIP-seq samples

The bedtools Fisher tool was used to compare the consensus ATAC-seq and ChIP-seq peaklists. Bedtools reldist was used to compute the relative distance distribution between ChIP-seq peaks and ATAC-seq peaks. For comparison of whole-genome signal, genomic bins of 100,000 bp were generated using bedtools makewindows followed by bedtools map, and similarities in the genome-wide coverage were then computed using Pearson correlation.

### Dose-response curves

Cells were plated at 4000 cells/well in NS media into laminin-poly-l-ornithine coated 96-well plates (Corning). The compounds MI-3, RGFP-966, and AZD6102 (Selleckchem) were tested at different concentrations (range: 2 μM to 8 nM in serial dilutions) with six technical replicates per dose, and a DMSO control. Cell viability was assessed on day 7. Alamar blue (Thermo Fisher Scientific, Cat# DAL1025) was added and cells were incubated at 37 °C in the dark for 4 h. Fluorescence was measured on the Spectramax spectrophotometer and was normalized to the DMSO control.

### Flow cytometry

#### Cell preparation and harvesting

Glioma cells grown on PLO-laminin plates were passaged with Accutase (StemCell Technologies) and resuspended in PBS.

#### CD44 flow cytometry

Cells were stained using an APC-conjugated anti-human anti-CD44 antibody (REA690; 1:200 concentration) for 20 min at 4 °C on a rotator. Cells were washed twice in PBS and passed through a cell strainer top tube to remove any clumps (Corning 352235).

#### Cell cycle analysis

Cells were stained at 1:1000 with Dye Cycle Violet (Life Technologies V35003) and incubated at 37 °C for 30 min, as per manufacturer protocols.

#### Flow cytometry data acquisition

Data was acquired on an Attune NxT flow cytometer (Invitrogen) at the Cumming School of Medicine Flow Cytometry Facility and data analysis were performed using FlowJo and CytoExploreR (https://github.com/DillonHammill/CytoExploreR). Gating was performed by gating on FSC-A and SSC-A to exclude cellular debris, followed by gating FSC-A and FSC-H to select single cells and remove doublets, followed by gating of GFP-positive cells compared to a cell-type matched GFP-negative control (Figure S7a–d). All experiments were performed in biological triplicate.

### Immunofluorescence high-content drug screening

#### Screen procedure

G523 cells were plated at a density of 5000 cells per well in a 96-well optical plate coated with PLO-laminin as per protocol. An epigenetic drug library (Z195677-L1900; Selleckchem) was added at 1 μM to plates in triplicate, with DMSO used as a control. Cells were incubated for 10 days and fixed in 4% PFA for 10 min, followed by storage at 4 °C until imaging.

#### Immunocytochemistry and imaging

Plates were blocked in 5% BSA in PBS with 0.1% Triton X-100 for 1 h at room temperature. Staining was performed overnight at 4 °C in the same media with the anti-rabbit macroH2A2 antibody (Invitrogen; PA5-57437) at a dilution of 1:250, and a fluorescently conjugated secondary antibody (Invitrogen Goat anti-rabbit IgG A568), and a DAPI counterstain (1:1000; Thermo Fisher #62248). Washes were performed using PBS with 0.1% Triton X-100. Samples were left in the plates in PBS and imaged using the GE InCell 6000 with a 60x objective in three different planes of section.

#### Image analysis

Images for each well were stitched together using Fiji and the grid/collection stitching plugin^121^. The DAPI channel was used to generate a list of nuclei and Fiji was used to measure intensity for both DAPI and the GFP channel using an automated custom script. Data was then further analysed using R, where data for all compounds on one plate was pooled together, and k-means clustering using brightness and shape parameters was used to stratify live/dead cells and mH2A2 positive or negative cells. These categories were then used to separate cells in each well as alive and mH2A2-negative, alive and mH2A2-positive, and dead for subsequent analysis. Percentages of mH2A2-positive cells were then compared to DMSO control to calculate a fold change.

### Primer and hairpin sequences

All primer sequences and hairpins used in this study have been included as a Supplementary Data file.

### General statistical analysis and data visualization

Unless otherwise specified, pairwise significance analyses were performed using a two-tailed *T*-test with Welch’s correction. All boxplots are plotted with a line for the median, hinges at 25th and 75th percentiles, and whiskers at 1.5 x IQR. Plots were generated using GraphPad Prism 9.0 or R studio with ggplot2.

### Reporting summary

Further information on research design is available in the Nature Portfolio Reporting Summary linked to this article.

## Supplementary information

Supplementary Information

Reporting Summary

Description of Additional Supplementary Files

Supplementary Data 1

## Data Availability

All RNA-seq, ATAC-seq, and ChIP-seq datasets generated in this study were deposited to the Gene Expression Omnibus (geo) with series IDs GSE149303, GSE149324, and GSE149334 and GSE212086. The scATAC-seq data of adult GBM has been published and is available in GEO (accession: GSE139136)^71^. Survival data were previously published^59–61^ and is accessible via the r2 data portal [https://hgserver1.amc.nl/cgi-bin/r2/main.cgi]. The single-cell GBM data used in this study was previously published and is available at GSE131918 and on the Broad Single Cell Portal [https://singlecell.broadinstitute.org/single_cell/study/SCP503/gradient-of-developmental-and-injury-reponse-transcriptional-states-define-functional-vulnerabilities-underpinning-glioblastoma-heterogeneity]^8,16^. Data on *MACROH2A2* expression in mouse and human brain has been published and is available at Brain RNA-seq [https://www.brainrnaseq.org/] and DropViz [http://dropviz.org/]^65,66^. Data on *MACROH2A2* expression in xenografts and multiregional samples was obtained from GSE139261 and GSE117891, respectively. The remaining data were available within the Article, Supplementary Information, or Source Data file. Source data are provided in this paper.

## Source data

Source data

## Code availability

All scripts used for analysis are available from the authors upon request.
