# Supplementary material for: macroH2A2 antagonizes epigenetic programs of stemness in glioblastoma
Source: Nat Commun. 2023 May 27;14:3062. doi: 10.1038/s41467-023-38919-2 (PMC10224928; doi:10.1038/s41467-023-38919-2)
Supplement: Supplementary file 1 — Supplementary Information [file 41467_2023_38919_MOESM1_ESM.pdf]

SUPPLEMENTARY INFORMATION

Supplementary Table 1. Clinical and molecular information for tumour samples used in this study.

Specimen	Age (years)	Sex	Diagnosis	IDH status	ATRX status	Chr 7	Chr 10	Amplifications
G523	Average: 60.7<br><br>Median: 60.5<br><br>Range: 50 - 73	Male, n = 5<br><br>Female, n = 1	glioblastoma, WHO grade 4	wildtype	N/A	Gain of 7p, 7q	equivocal	EGFR, CDK4, MDM2
GSC2			glioblastoma, WHO grade 4	wildtype	Retained	Gain of 7p, 7q	Loss of chr10p, chr10q	EGFR
GSC3			glioblastoma, WHO grade 4	wildtype	Retained	Gain of 7p, 7q	Loss of chr10p, chr10q (subclonal)	none
SM4447			glioblastoma, WHO grade 4	wildtype	Retained	N/A	N/A	N/A
SM4691			glioblastoma, WHO grade 4	wildtype	Retained	N/A	N/A	N/A
SM4491			glioblastoma, WHO grade 4	wildtype	Retained	N/A	N/A	N/A

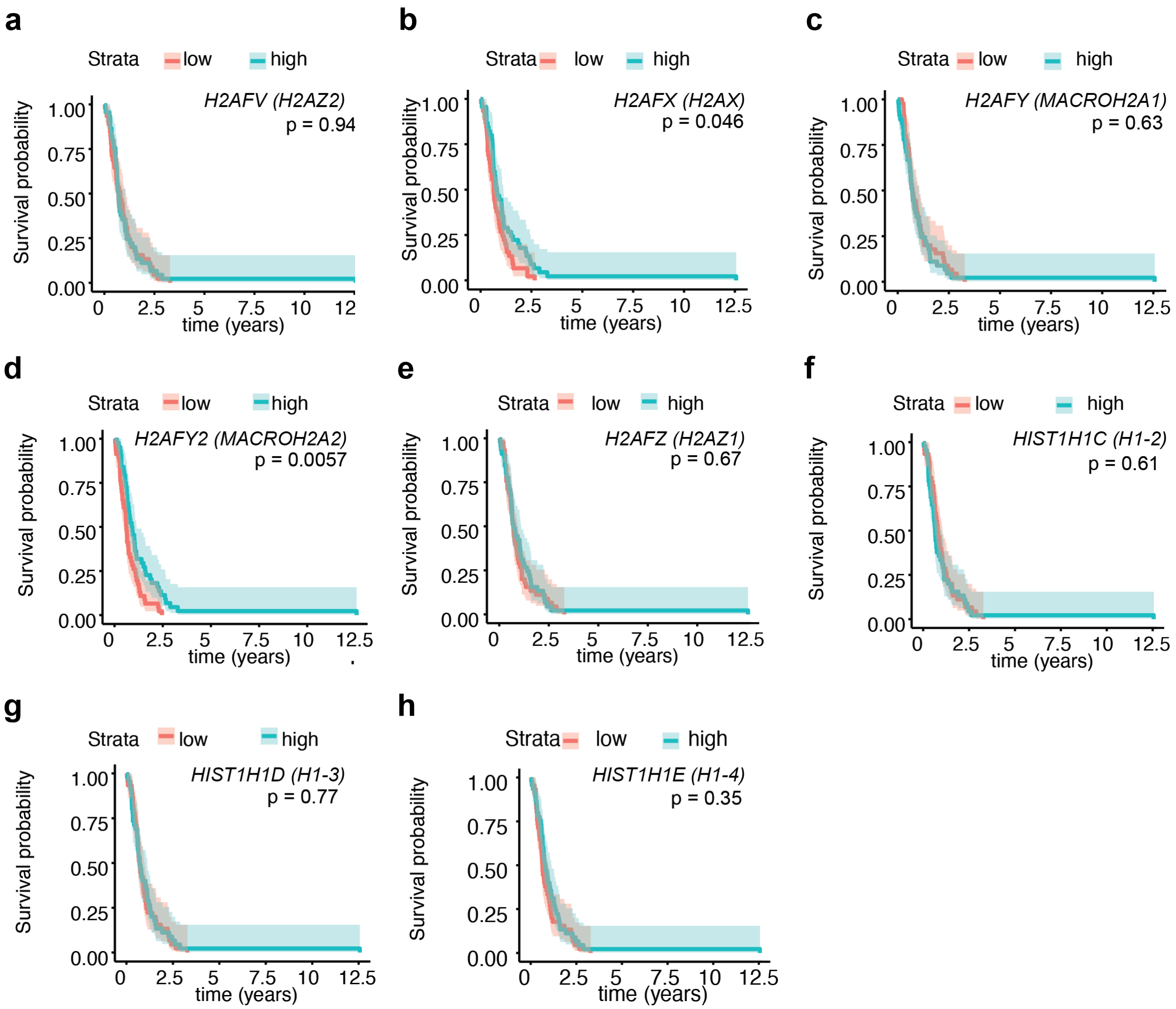

Supplementary Figure S1. (a-h) Survival analyses of the Gravendeel et al[1] dataset for histone variants **(a)** *H2AFV (H2AZ2)*, **(b)** *H2AFX (H2AX)*, **(c)** *H2AFY (MACROH2A1)*, **(d)** *H2AFY2 (MACROH2A2)*, **(e)** *H2AFZ (H2AZ1)*, **(f)** *HIST1H1C (H1-2),* **(g)** *HIST1H1D (H1-3)*, **(h)** *HIST1H1E (H1-4)*. P values computed by log-rank test.

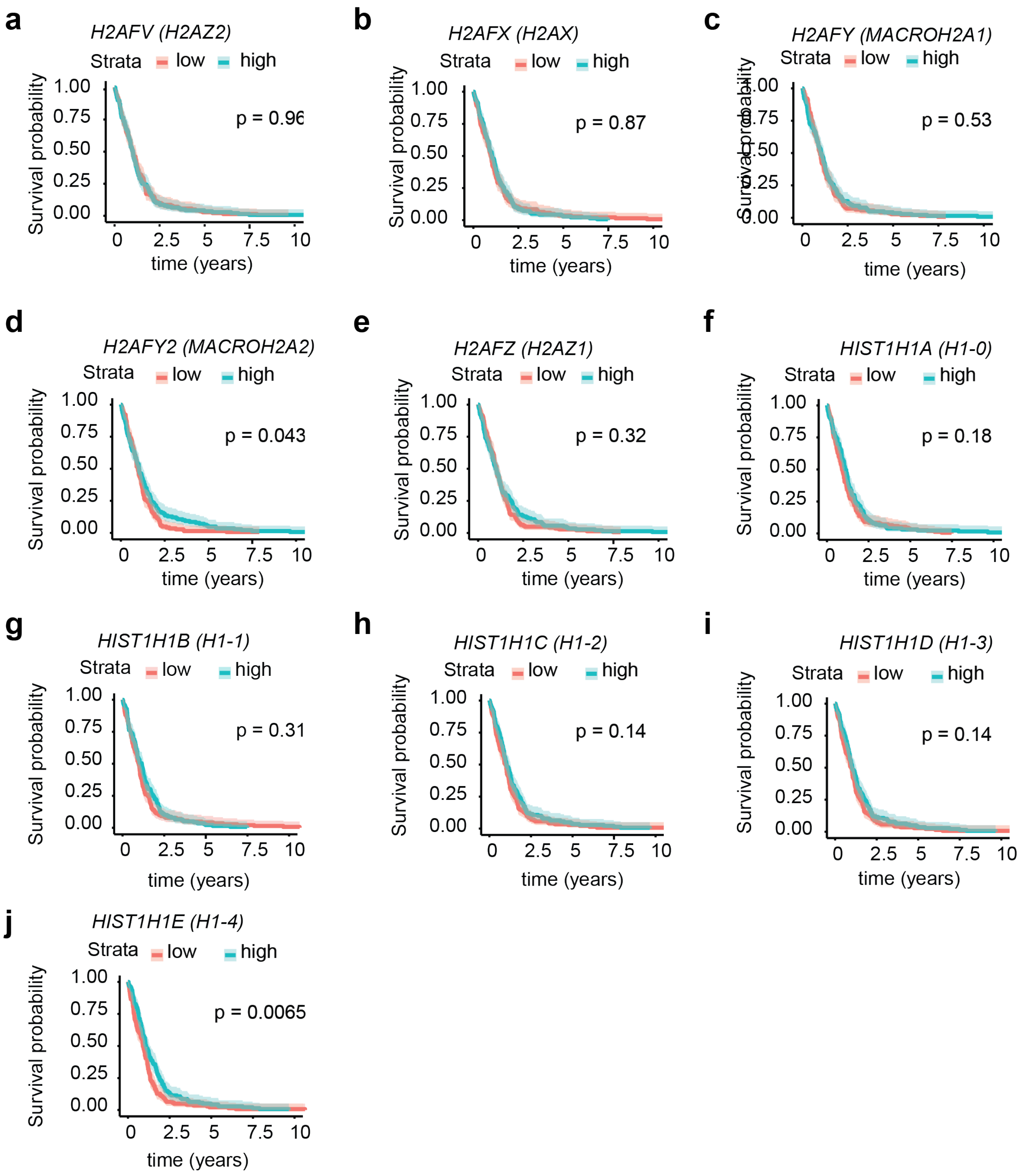

Supplementary Figure S2. (a-j) Survival analyses of the TCGA high-grade glioma dataset[2,3] for histone variants **(a)** *H2AFV (H2AZ2)*, **(b)** *H2AFX (H2AX)*, **(c)** *H2AFY (MACROH2A1)*, **(d)** *H2AFY2 (MACROH2A2)*, **(e)** *H2AFZ (H2AZ1)*, **(f)** *HIST1H1A (H1-0),* **(g)** *HIST1H1B (H1-1)*, **(h)** *HIST1H1C (H1-2)*. **(i)** *HIST1H1D (H1-3),* **(j)** *HIST1HE (H1-5)*. P values computed by log-rank test.

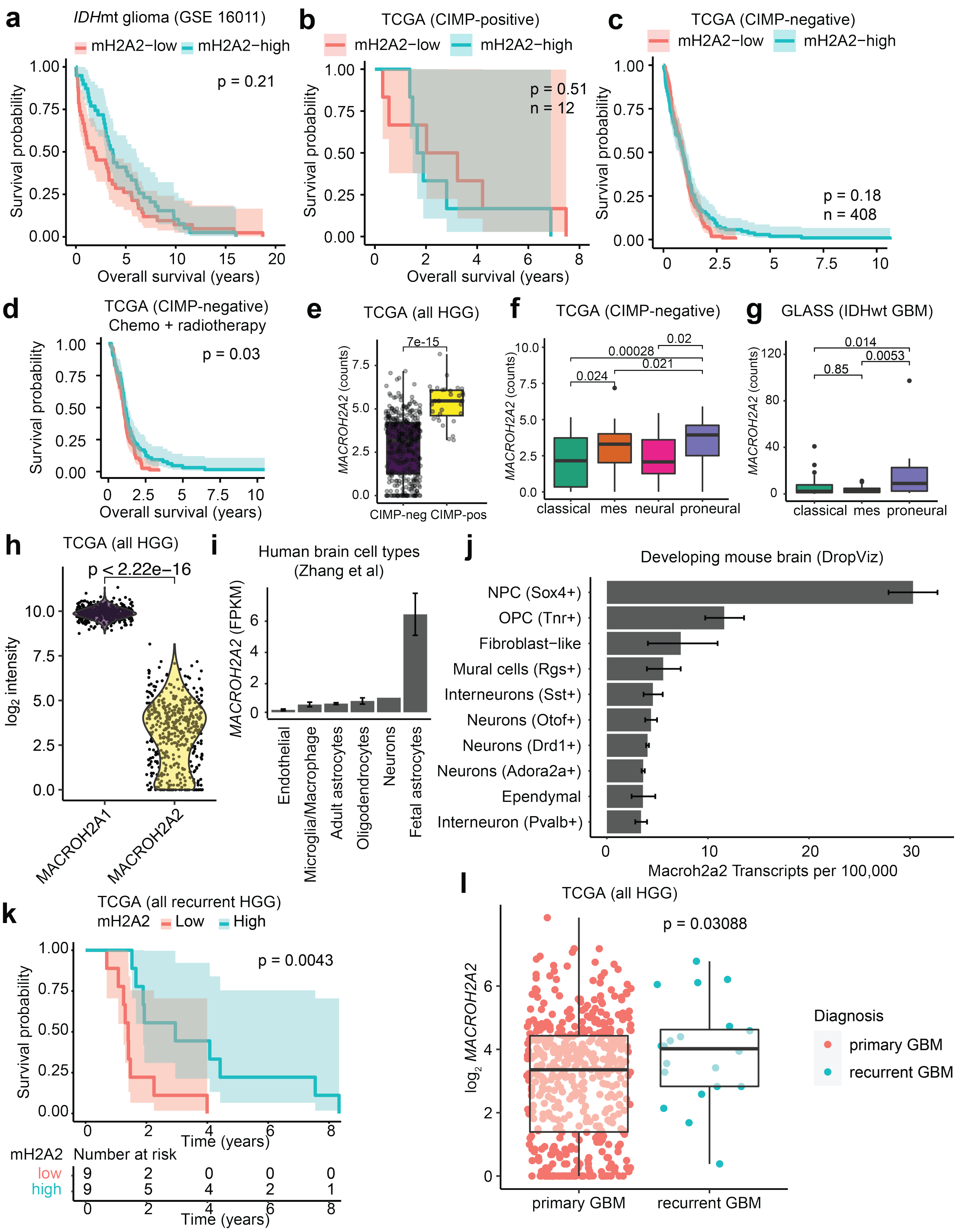

Supplementary Figure S3.

(a) Overall survival for *IDH* mutant gliomas in GSE 16011 stratified by median *MACROH2A2* (mH2A2). P value calculated by log-rank test. Shaded region represents 95% confidence interval.

(b) Overall survival for CIMP-positive mutant gliomas in the TCGA high-grade glioma cohort stratified by median *MACROH2A2* (mH2A2). P value calculated by log-rank test. Shaded region represents 95% confidence interval.

(c) Overall survival for CIMP-negative mutant gliomas in the TCGA high-grade glioma cohort stratified by median *MACROH2A2* (mH2A2). P value calculated by log-rank test. Shaded region represents 95% confidence interval.

(d) Overall survival for CIMP-negative mutant gliomas treated with chemotherapy and radiotherapy in the TCGA high-grade glioma cohort stratified by median *MACROH2A2* (mH2A2). P value calculated by log-rank test. Shaded region represents 95% confidence interval.

(e) MACROH2A2 levels in CIMP-negative and CIMP-positive tumours in the TCGA high-grade glioma cohort. P value by two-tailed unpaired T test with Welch's correction. Boxplot line represents median, hinges at 25th and 75th percentiles, and whiskers at 1.5 x IQR.

(f) MACROH2A2 expression in CIMP-negative tumours in TCGA high-grade glioma cohort separated by Verhaak transcriptional subtype. P value by two-tailed unpaired T test with Welch's correction.. Boxplot line represents median, hinges at 25th and 75th percentiles, and whiskers at 1.5 x IQR.

(g) MACROH2A2 expression in GLASS consortium primary IDH-wildtype GBM separated by Verhaak transcriptional subtype. P value by two-tailed unpaired T test with Welch's correction. Boxplot line represents median, hinges at 25th and 75th percentiles, and whiskers at 1.5 x IQR.

(h) Expression data for *MACROH2A1* and *MACROH2A2* in the TCGA high-grade glioma cohort (CIMP-positive and CIMP-negative). P value by two-tailed unpaired T test with Welch's correction.

(i) Expression of *MACROH2A2* in immunopanned human brain cell types.[4] Error bars represent standard error.

(j) Expression of *Macroh2a2* in different cell types in the mouse brain[5]. Error bars represent upper and lower 95% confidence interval.

(k) Kaplan-Meier survival status for patients with recurrent high-grade glioma in TCGA cohort – CIMP-positive and CIMP-negative (shaded region represents 95% confidence interval; p value calculated by log-rank test).

(l) Comparison of expression levels for *MACROH2A2* in TCGA data (CIMP-positive and CIMP-negative) for untreated versus treated primary glioblastoma (p value: two-tailed T test with Welch's correction; whiskers represent 95% confidence interval). Boxplot line represents median, hinges at 25th and 75th percentiles, and whiskers at 1.5 x IQR.

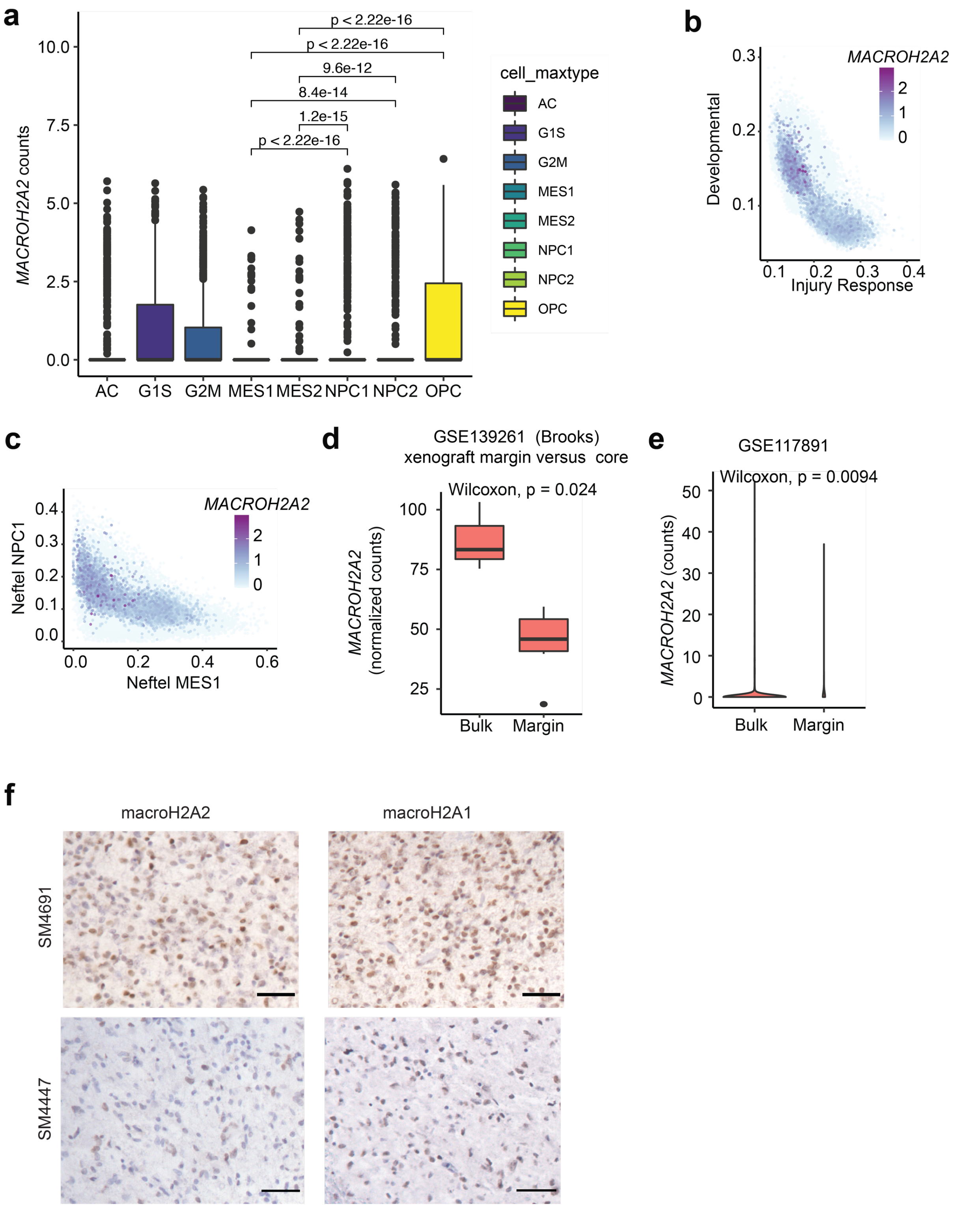

Supplementary Figure S4. (a) *MACROH2A2* counts in different cell subsets in the Neftel et al[6] dataset (P values; unpaired two-tailed T-test with Welch's correction). Boxplot line represents median, hinges at 25th and 75th percentiles, and whiskers at 1.5 x IQR. **(b-c)** *MACROH2A2* counts in Richards et al[7] dataset plotted against **(b)** developmental and injury response axes and **(c)** Neftel NPC1 and MES1 axes **(c)**. Comparison of *MACROH2A2* expression levels at tumour

bulk versus margin regions in **(d)** Brooks et al xenografts[8] (GSE139261) [Boxplot line represents median, hinges at 25th and 75th percentiles, and whiskers at 1.5 x IQR; P value by Wilcoxon test] and **(e)** individual tumour cells from Yu et al[9] regionalized scRNA-seq data from gliomas (GSE117891; P value: Wilcoxon test). **(f)** Immunohistochemistry of macroH2A2 and macroH2A1 in two patient tumours (scale bar 50 microns).

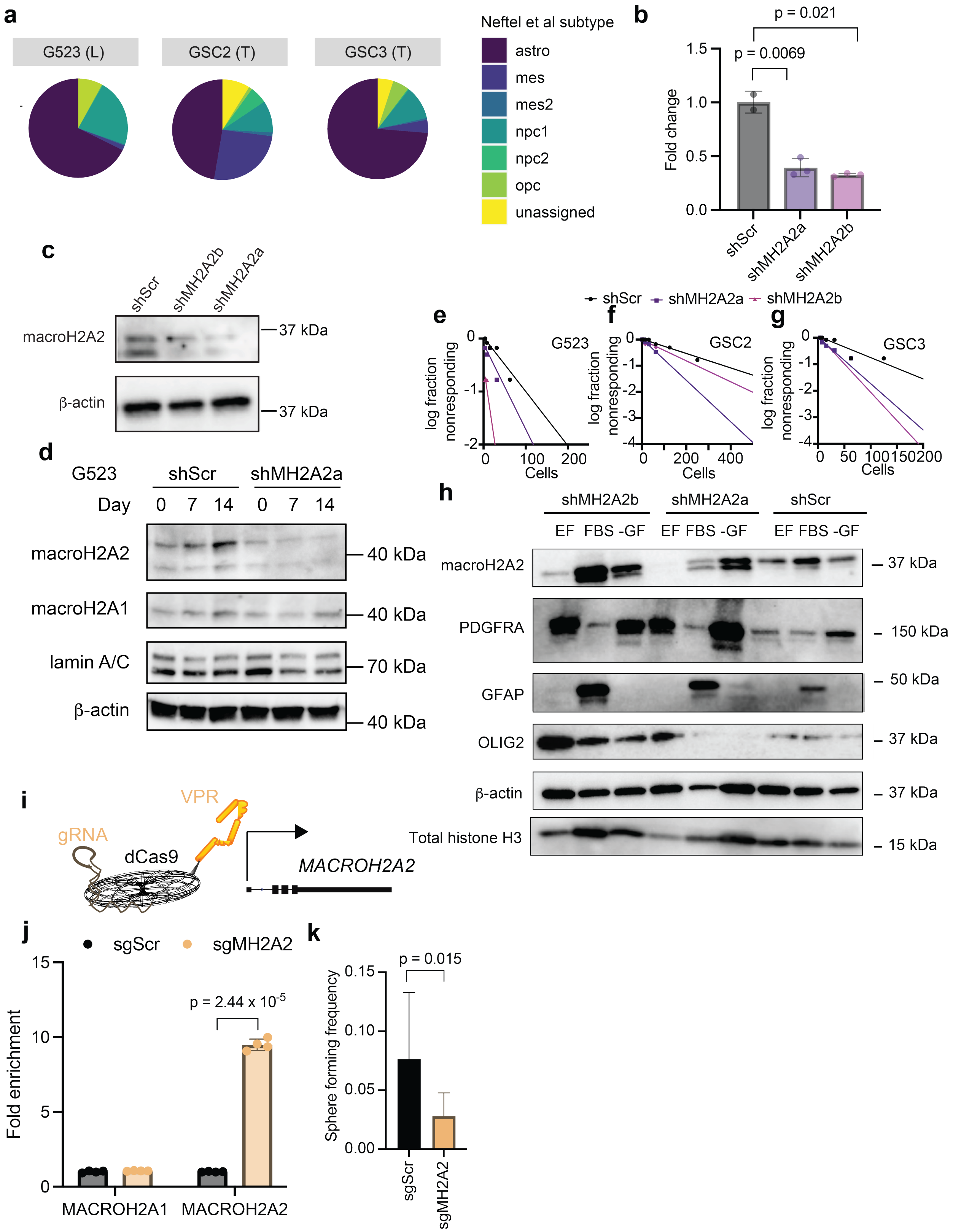

a
G523 (L)
GSC2 (T)
GSC3 (T)
Neftel et al subtype
astro
mes
mes2
npc1
npc2
opc
unassigned
b
p = 0.021
p = 0.0069
Fold change
1.5
1.0
0.5
0.0
shScr
shMH2A2a
shMH2A2b
c
shScr
shMH2A2b
shMH2A2a
macroH2A2
β-actin
37 kDa
37 kDa
d
G523
shScr
shMH2A2a
Day
0 7 14
0 7 14
macroH2A2
macroH2A1
lamin A/C
β-actin
40 kDa
40 kDa
70 kDa
40 kDa
shScr
shMH2A2a
shMH2A2b
e
f
g
G523
GSC2
GSC3
log fraction nonresponding
Cells
h
shMH2A2b
shMH2A2a
shScr
EF FBS -GF
EF FBS -GF
EF FBS -GF
macroH2A2
PDGFRA
GFAP
OLIG2
β-actin
Total histone H3
37 kDa
150 kDa
50 kDa
37 kDa
37 kDa
15 kDa
i
VPR
gRNA
dCas9
MACROH2A2
j
sgScr
sgMH2A2
p = 2.44 x 10-5
Fold enrichment
15
10
5
0
MACROH2A1
MACROH2A2
k
p = 0.015
Sphere forming frequency
0.15
0.10
0.05
0.00
sgScr
sgMH2A2

Supplementary Figure S5. (a) Characterization of cell subtypes in primary tumours based on published scRNA-seq (G523)[7] and scATAC-seq datasets. **(b)** RT-qPCR validation of *MACROH2A2* transcript levels at 48 hours post-induction (center represents overall per-condition mean; 3 biological replicates/condition; error bars represent standard error; p value by unpaired two-tailed T-test). **(c)** macroH2A2 protein levels after two weeks of induction in two hairpins. Experiment repeated three times. **(d)** Western blot of macroH2A2 and macroH2A1 after 7 or 14 days of activation with doxycycline. Experiment repeated two times.
(e-g) Log fraction non-responding plots for limiting dilution assays on **(e)** G523, **(f)** GSC2 and **(g)** GSC3 cells.
(h) Western blot showing differentiation marker expression in G523 cells after 7 days of MACROH2A2 knockdown and 7 days of culture under stem (EF) or differentiation conditions [FBS – 1% FBS; -GF – growth factor withdrawal (no EGF or FGF)]. Experiment performed twice.
(i) Schematic of dCas9-based overexpression model.
(j) RT-qPCR validation of transcript levels of *MACROH2A1* and *MACROH2A2* after transfection with sgRNAs (center: overall per-condition mean; 3 biological replicates/condition; error bars represent standard error; p value by unpaired two-tailed T-test).
(k) Sphere forming frequency of sgMH2A2 versus Scramble sgRNA treated cells. P value was determined by Chi-square test with the tool ELDA (see Methods). Center: point estimate of sphere formation potential. Error bars: 95% confidence interval. Statistics from 6 technical replicates.

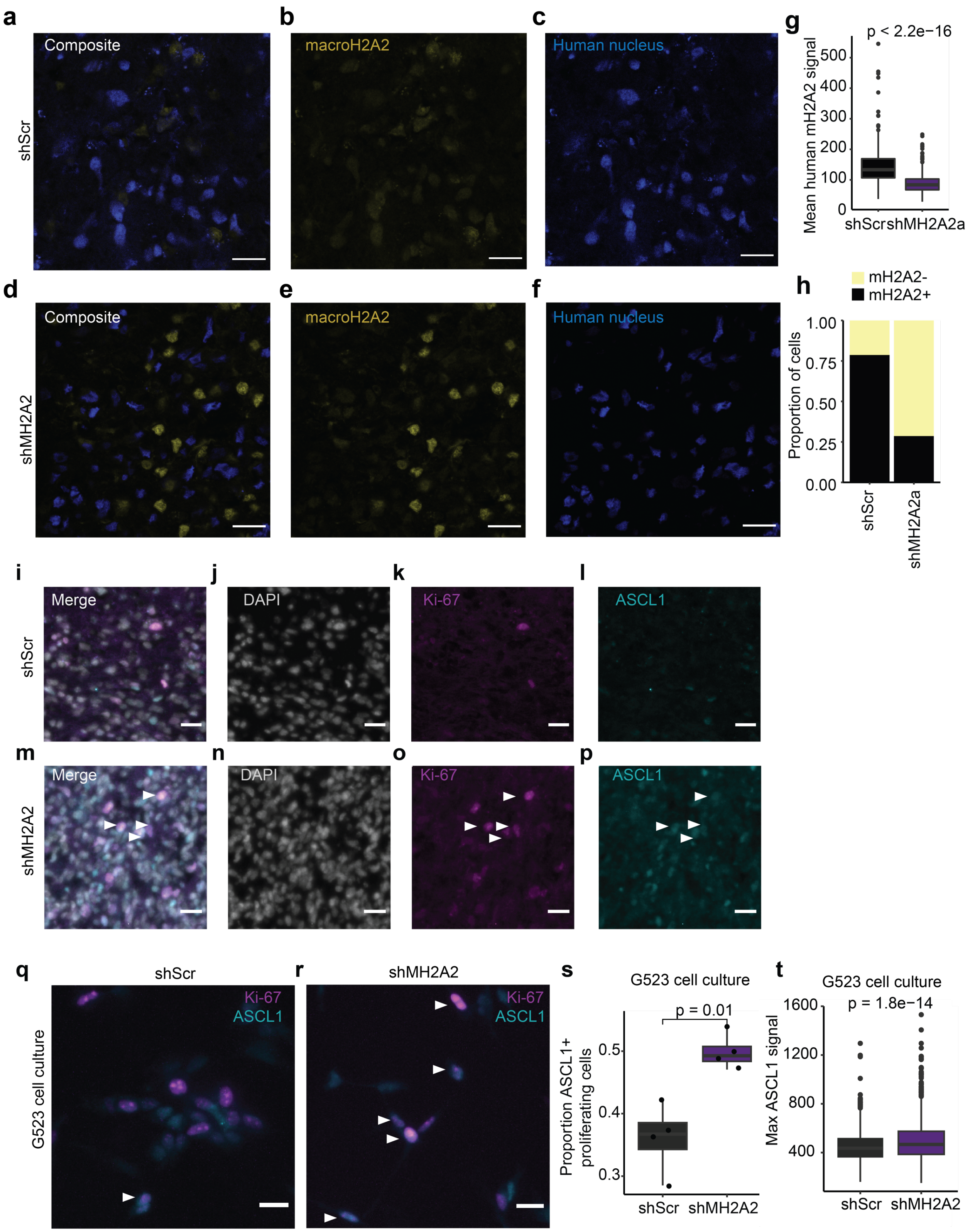

Supplementary Figure S6.
(a-f) Representative confocal microscopy images of macroH2A2 and human nucleus stained cells in shScr control xenografts **(a-c)** and knockdown xenografts **(d-f)** [scale bar: 20 microns].

(g-h) Quantification of human macroH2A2 signal **(g)** and proportion of positive cells **(h)** by confocal microscopy over a 20x field in scramble and knockdown cells (p values: unpaired two-tailed T-test); repeated over two fields. Boxplot line represents median, hinges at 25th and 75th percentiles, and whiskers at 1.5 x IQR. **(i-p)** Representative widefield microscopy images of Ki-67 and ASCL1 colabeling in control **(i-l)** and knockdown **(m-p)** xenograft mice. Scale bar: 25 microns. Experiment repeated in at least 2 mice per condition. **(q-r)** Immunocytochemistry for Ki-67 and ASCL1 in G523 control **(q)** or shMH2A2 **(r)** cells after 1 week of induction. Experiment repeated on two biological replicates. Scale bar: 25 microns. **(s)** Comparison of ASCL1+ proliferating cells between control and knockdown conditions. P value: unpaired two-tailed T test with Welch's correction. Boxplot line represents median, hinges at 25th and 75th percentiles, and whiskers at 1.5 x IQR. **(t)** Maximum ASCL1 signal intensity per cell in control versus shMH2A2 cells. P value: unpaired two-tailed T-test with Welch's correction. Boxplot line represents median, hinges at 25th and 75th percentiles, and whiskers at 1.5 x IQR.

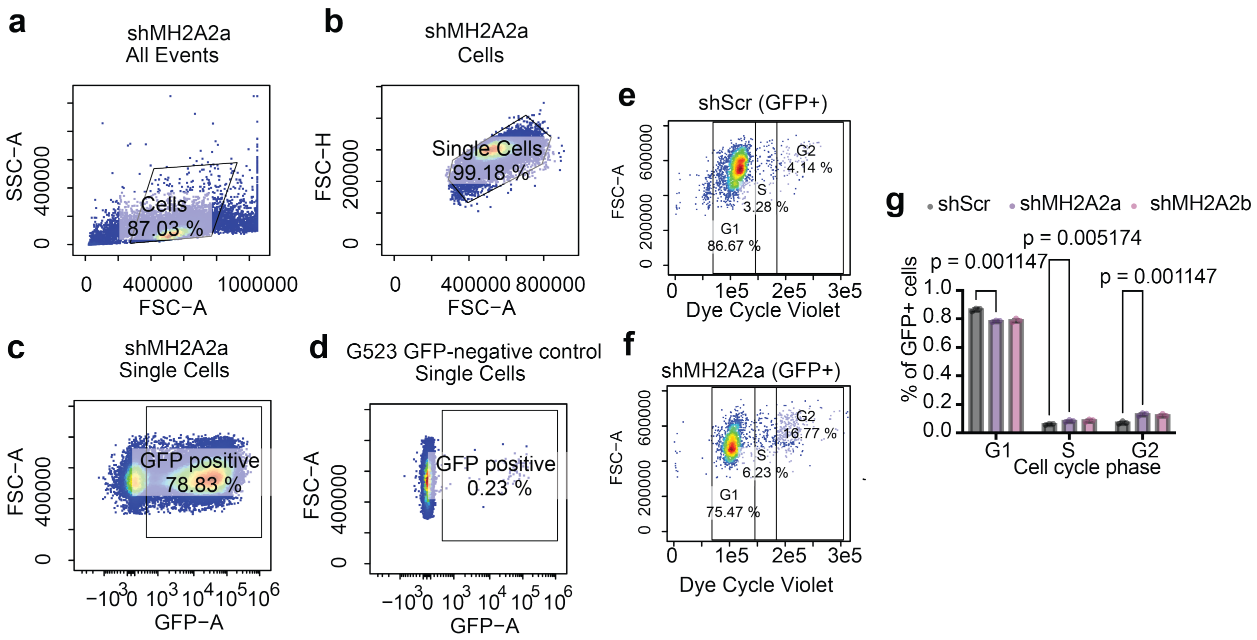

Supplementary Figure S7

(a-d) Flow cytometry analysis showing gating strategy for flow cytometry experiments of CD44 and cell cycle analysis on knockdown cells induced for 7 days. **(c-d)** Example of GFP positivity in knockdown cells induced for 7 days **(c)** versus uninduced control **(d)**

(e-g) Cell cycle analysis of G523 cells by Dye Cycle Violet: representative traces of control and knockdown cells **(e-f)** and quantification **(g)** of biological replicates (n = 3). P values: unpaired two-tailed T-test with Welch's correction. Error bars represent standard deviation.

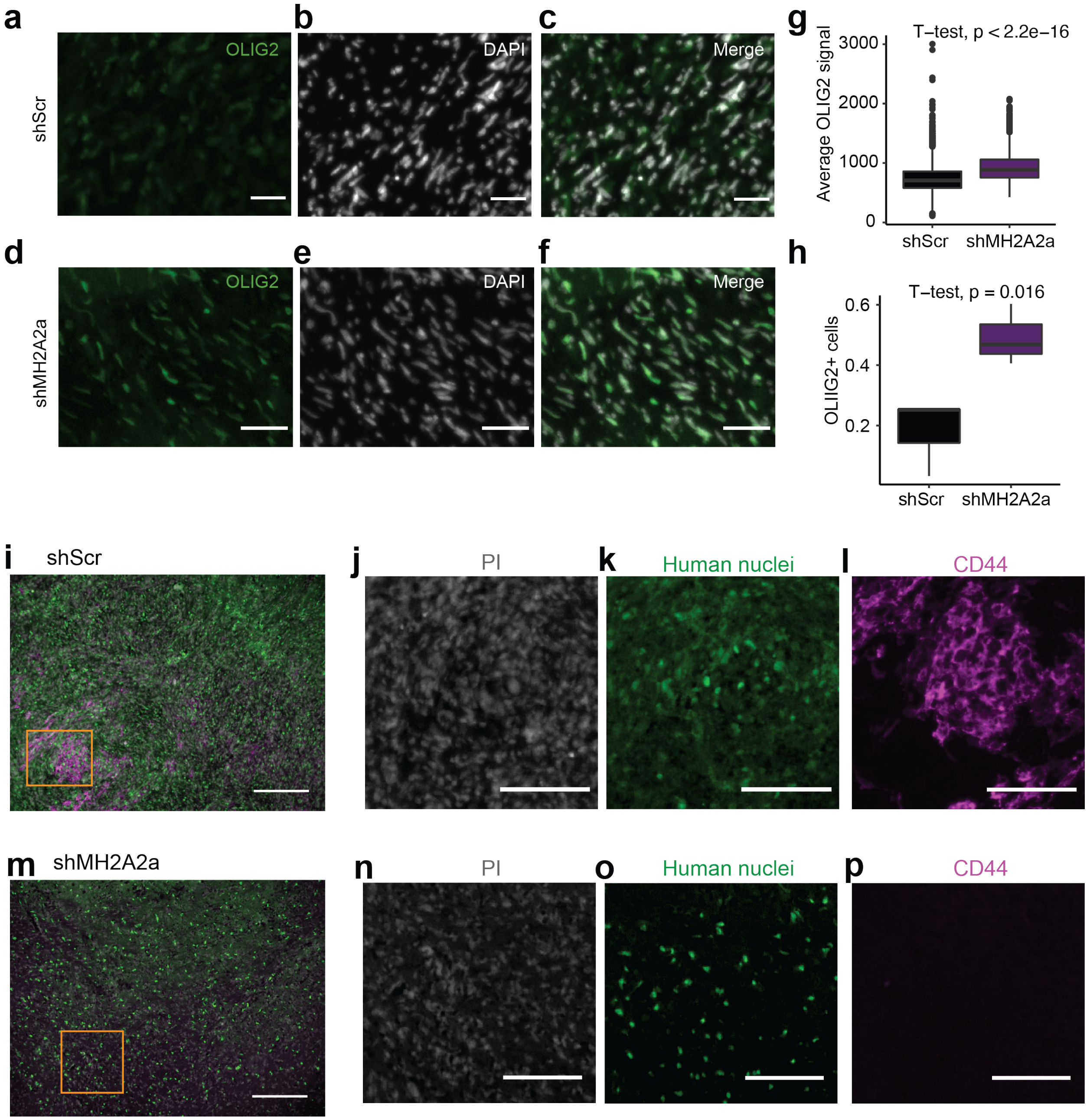

Supplementary Figure S8.

(a-f) Representative images of shScr **(a-c)** and shMH2A2a **(d-f)** xenografts stained for OLIG2. **(g)** Quantification of average OLIG2 signal (p value calculated by unpaired two-tailed T-test). Boxplot line represents median, hinges at 25th and 75th percentiles, and whiskers at 1.5 x IQR. **(h)** Proportion of OLIG2-positive cells in control versus knockdown tumours (p value calculated by unpaired two-tailed T-test). Quantification performed over 3 10x fields. Scale bar: 50 microns. Boxplot line represents median, hinges at 25th and 75th percentiles, and whiskers at 1.5 x IQR. **(i-l)** Fluorescence immunohistochemistry of control xenografts stained for human nuclear antigen, propidium iodide, and CD44**. (i)** represents composite image (scale bar 200 mm), and **(j)-(l)** represent magnified single channel images of the inset area in orange (scale bar 100 mm).

(m-p) Fluorescence immunohistochemistry of shMH2A2a xenografts stained for human nuclear antigen, propidium iodide, and CD44**. (m)** represents composite image (scale bar 200 mm), and **(n)-(p)** represent magnified single channel images of the inset area in orange (scale bar 100 mm). Experiments in **(i)-(p)** repeated twice on three independent animals.

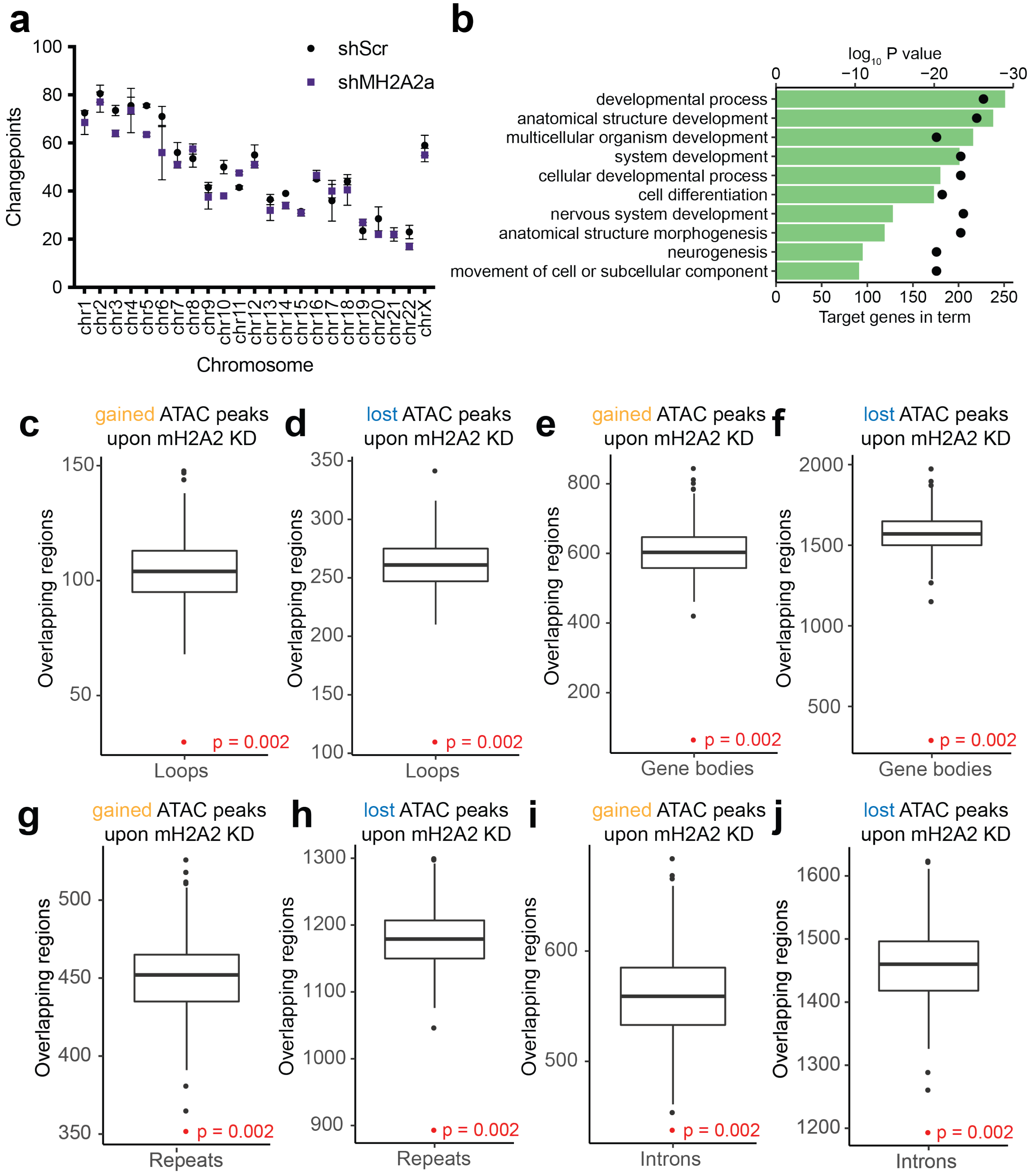

Supplementary Figure S9.
(a) Changepoint analysis of knockdown (shMH2A2a) versus control cells. Error bars represent standard deviation.
(b) GO process terms enriched in peaks lost upon macroH2A2 knockdown. P value: hypergeometric test.

(c-j) Permutation analysis of regions with at least 1.5 fold log_2 change in accessibility upon *MACROH2A2* knockdown with **(c-d)** DNA loop boundaries (Johnston et al 2019); **(e-f)** Gene bodies; **(g-h)** repeat regions from the RepeatMasker database; **(i-j)** Introns. Regions of accessibility gain and loss were analysed separately. P value by hypergeometric test from 500 permutations. Boxplot line represents median, hinges at 25th and 75th percentiles, and whiskers at 1.5 x IQR.

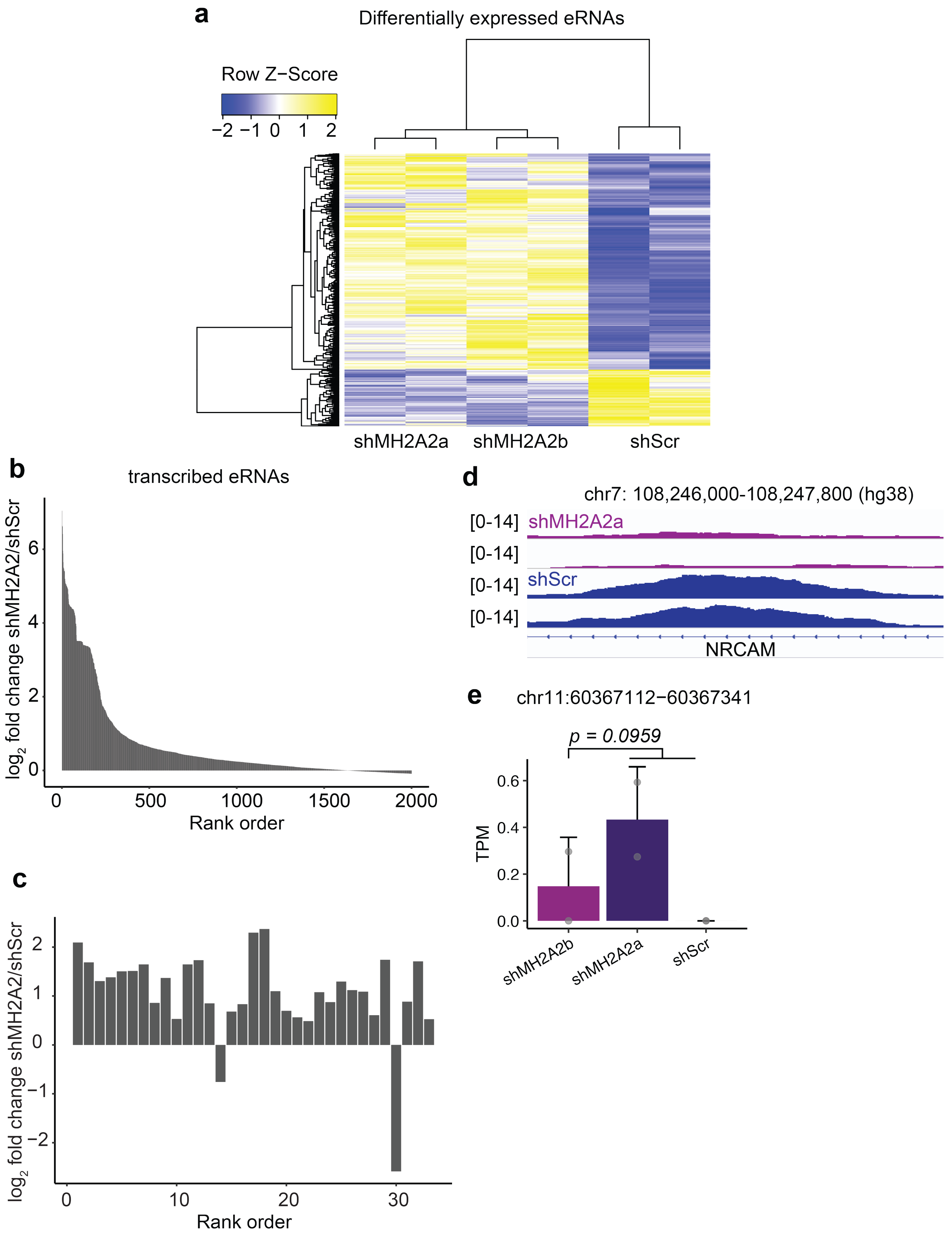

Supplementary Figure S10.

(a) Differential expression of top 200 differentially expressed GBM-specific putative eRNAs between *MACROH2A2* knockdown and control cells.

(b) Top 2000 differentially transcribed GBM-specific eRNAs between macroH2A2 knockdown and control cells, ranked by fold change.

(c) Close up of significantly differentially transcribed eRNA, ranked by p value.

(d) Example of a representative ATAC-seq enhancer peak lost upon macroH2A2 knockdown.

(e) Example of eRNA at an enhancer locus with increased accessibility. P value calculated by unpaired T test. Center: mean TPM per condition. Error bars represent standard deviation.

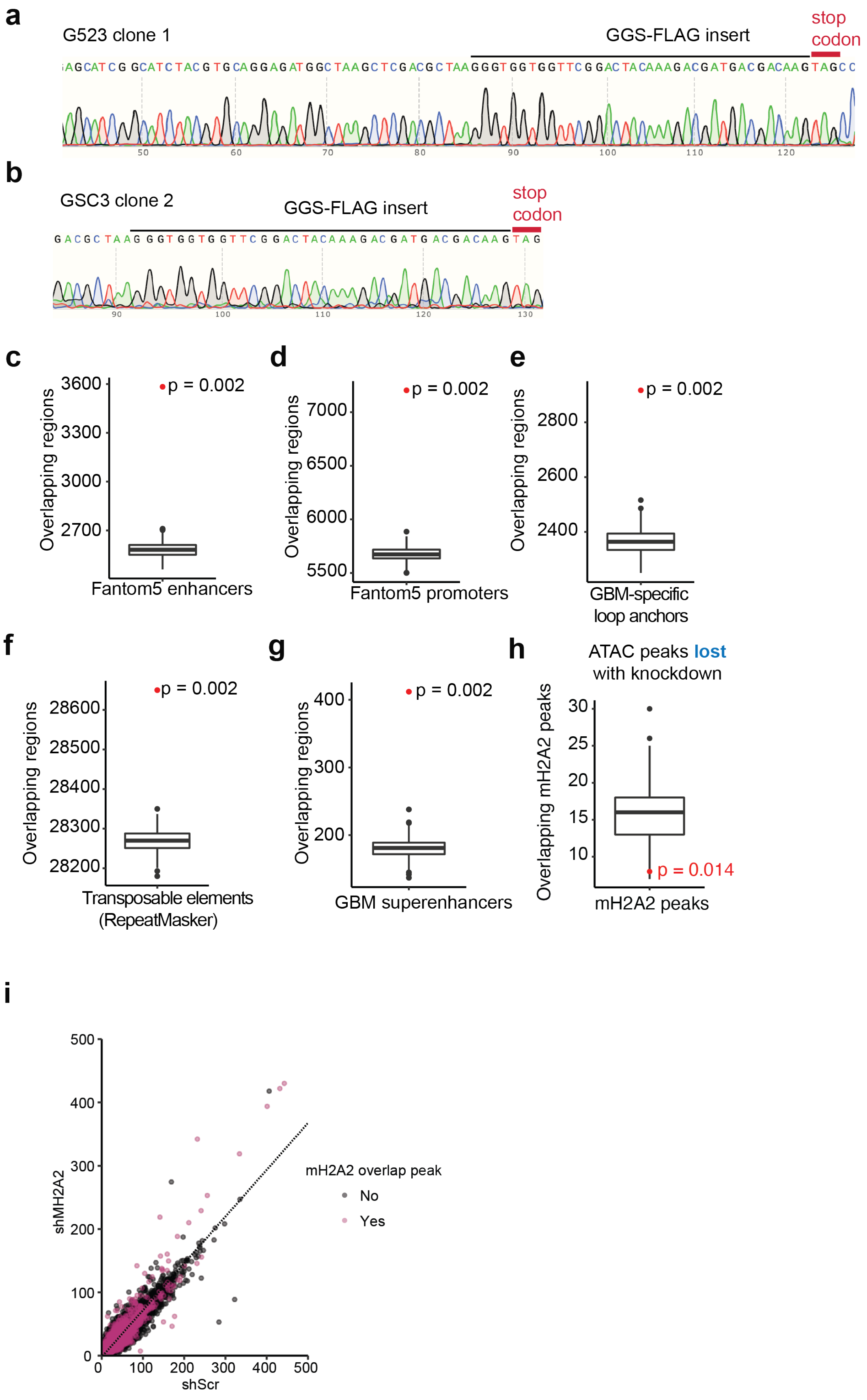
a
G523 clone 1
GGS-FLAG insert
stop codon
b
GSC3 clone 2
GGS-FLAG insert
stop codon
c
Overlapping regions
p = 0.002
Fantom5 enhancers
d
Overlapping regions
p = 0.002
Fantom5 promoters
e
Overlapping regions
p = 0.002
GBM-specific loop anchors
f
Overlapping regions
p = 0.002
Transposable elements (RepeatMasker)
g
Overlapping regions
p = 0.002
GBM superenhancers
h
ATAC peaks lost with knockdown
Overlapping mH2A2 peaks
p = 0.014
mH2A2 peaks
i
shMH2A2
shScr
mH2A2 overlap peak
No
Yes

Supplementary Figure S11.

(a-b) Sanger sequencing traces confirming in-frame FLAG insert in G523 **(a)** and GSC3 **(b)** clones. **(c-g)** Permutation analysis of macroH2A2 ChIP peaks with Fantom5 enhancers **(c)**, promoters **(d)**, GBM-specific loop anchors **(e)**, transposable elements **(f)**, and GBM superenhancers **(g). (h)** Permutation analysis of ATAC-seq peaks lost in shMH2A2a knockdown cells compared to macroH2A2 peaks. All P values by hypergeometric test, n = 500 permutations. Boxplot line represents median, hinges at 25th and 75th percentiles, and whiskers at 1.5 x IQR. **(i)** Scatterplot of ATAC peaks between control and knockdown cells colored by macroH2A2 overlap status.

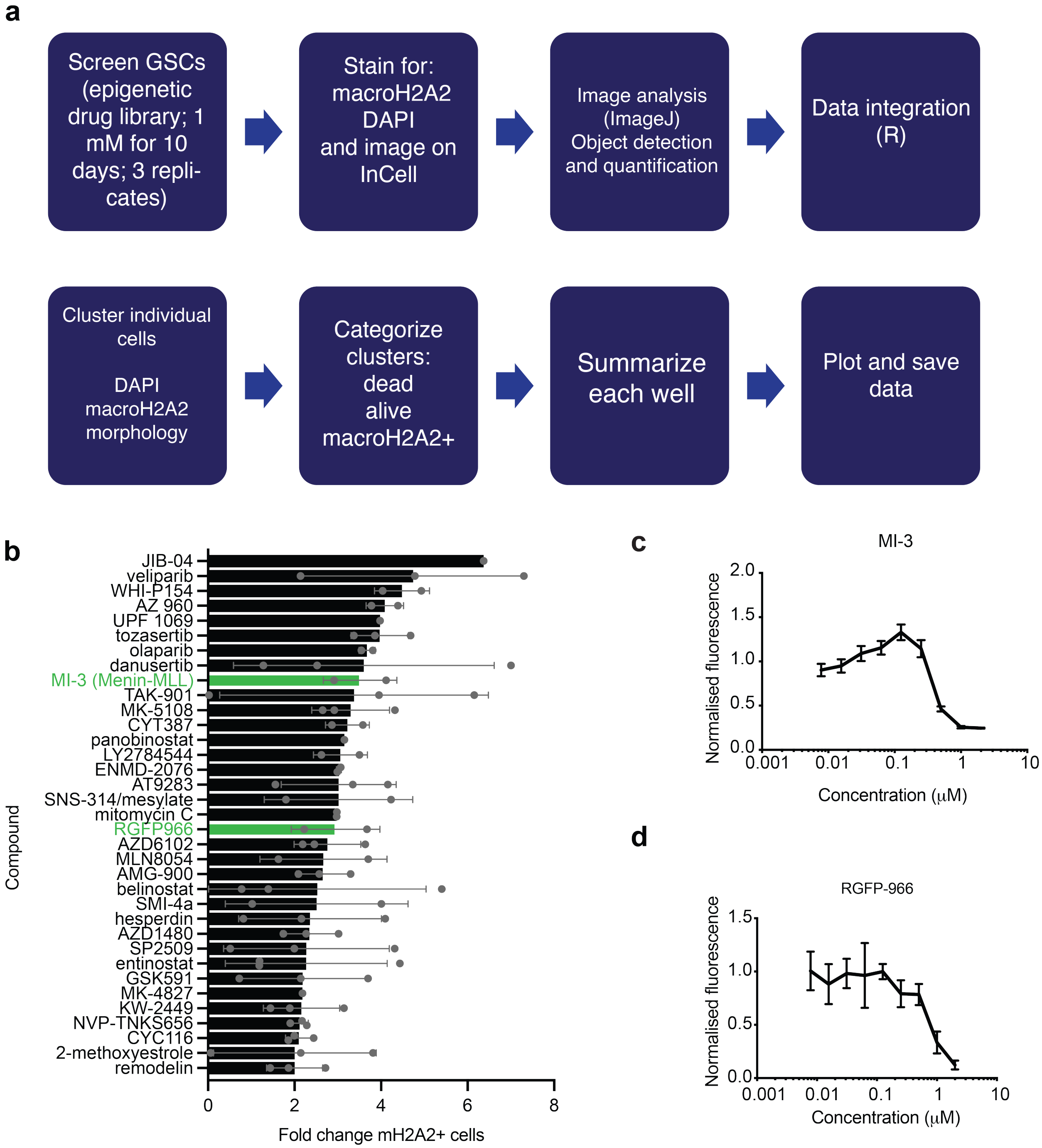

Supplementary Figure S12.
(a) Overview of high-content screening strategy.
(b) List of compounds showing more than 2-fold increase in macroH2A2 levels. Error bars represent standard deviations.
(c) Alamar blue *in vitro* dose-response curve for MI-3 in G523 cells. Six technical replicates per concentration. Error bars represent standard deviation. Experiment repeated two times.

(d) Alamar blue *in vitro* dose-response curve for RGFP-966 in G523 cells. Six technical replicates per concentration. Error bars represent standard deviation, and center represents mean signal at each concentration. Experiment repeated two times.

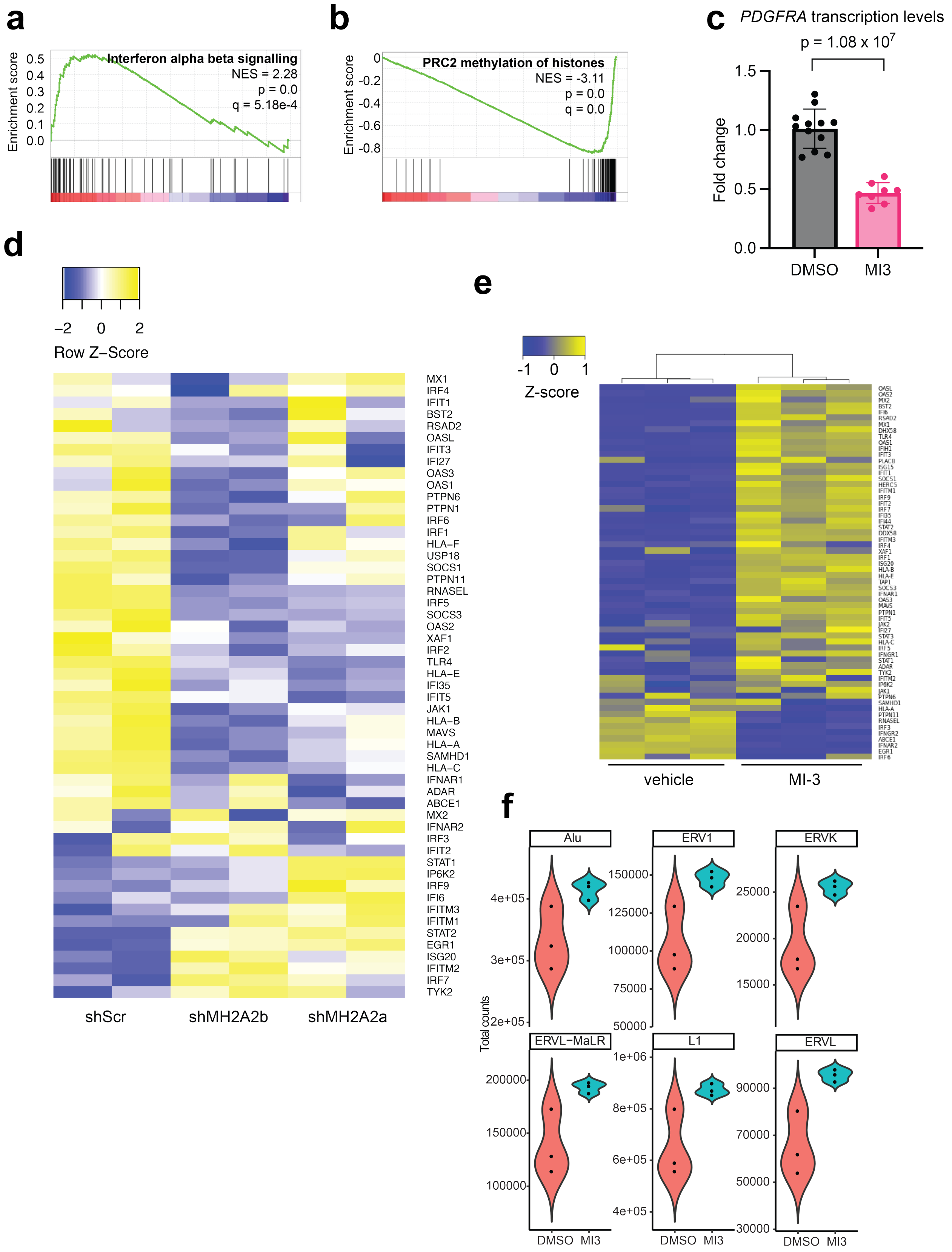

Supplementary Figure S13.

(a-b) GSEA results showing altered interferon signalling and methylation signatures upon MI-3 treatment compared to DMSO control. P value by hypergeometric test, q value by hypergeometric test with Benjamini-Hochberg correction.
(c) Change in *PDGFRA* transcription by qPCR upon MI-3 treatment. Three biological and three technical replicates per condition. Center represents mean fold change across all replicates. P value from unpaired two-tailed T-test. Error bars represent standard deviation. **(d)** Expression of ISGs in control versus macroH2A2 knockdown cells. **(e)** Expression of ISGs in MI3 and DMSO treated cells. **(f)** Expression of select repeat elements in DMSO versus MI-3 treated cells.

References:

1. Gravendeel, L. A. M. *et al.* Intrinsic gene expression profiles of gliomas are a better predictor of survival than histology. *Cancer Research* **69**, 9065–9072 (2009).
2. Brennan, C. W. *et al.* The somatic genomic landscape of glioblastoma. *Cell* **155**, 462–77 (2013).
3. Verhaak, R. G. W. *et al.* Integrated Genomic Analysis Identifies Clinically Relevant Subtypes of Glioblastoma Characterized by Abnormalities in PDGFRA, IDH1, EGFR, and NF1. *Cancer Cell* **17**, 98–110 (2010).
4. Zhang, Y. *et al.* Purification and Characterization of Progenitor and Mature Human Astrocytes Reveals Transcriptional and Functional Differences with Mouse. *Neuron* **89**, 37–53 (2016).
5. Saunders, A. *et al.* Molecular Diversity and Specializations among the Cells of the Adult Mouse Brain. *Cell* **174**, 1015–1030 (2018).
6. Neftel, C. *et al.* An Integrative Model of Cellular States, Plasticity, and Genetics for Glioblastoma. *Cell* **178**, 835-849.e21 (2019).
7. Richards, L. M. *et al.* Gradient of Developmental and Injury Response transcriptional states defines functional vulnerabilities underpinning glioblastoma heterogeneity. *Nature Cancer* **2**, 157–173 (2021).
8. Brooks, L. J. *et al.* The white matter is a pro-differentiative niche for glioblastoma. *Nature Communications* **12**, 2184 (2021).
9. Yu, K. *et al.* Surveying brain tumor heterogeneity by single-cell RNA-sequencing of multi-sector biopsies. *National Science Review* **7**, 1306–1318 (2020).
