# Supplementary material for: macroH2A2 antagonizes epigenetic programs of stemness in glioblastoma
Source: Nat Commun. 2023 May 27;14:3062. doi: 10.1038/s41467-023-38919-2 (PMC10224928; doi:10.1038/s41467-023-38919-2)
Supplement: Supplementary file 2 — Reporting Summary [file 41467_2023_38919_MOESM2_ESM.pdf]

nature portfolio

Corresponding author(s): Gallo, Marco

Last updated by author(s): 05/05/2023

Reporting Summary

Nature Portfolio wishes to improve the reproducibility of the work that we publish. This form provides structure for consistency and transparency in reporting. For further information on Nature Portfolio policies, see our Editorial Policies and the Editorial Policy Checklist.

Statistics

For all statistical analyses, confirm that the following items are present in the figure legend, table legend, main text, or Methods section.

n/a	Confirmed	
☐	☒	The exact sample size (n) for each experimental group/condition, given as a discrete number and unit of measurement
☐	☒	A statement on whether measurements were taken from distinct samples or whether the same sample was measured repeatedly
☐	☒	The statistical test(s) used AND whether they are one- or two-sided<br>*Only common tests should be described solely by name; describe more complex techniques in the Methods section.*
☐	☒	A description of all covariates tested
☐	☒	A description of any assumptions or corrections, such as tests of normality and adjustment for multiple comparisons
☐	☒	A full description of the statistical parameters including central tendency (e.g. means) or other basic estimates (e.g. regression coefficient) AND variation (e.g. standard deviation) or associated estimates of uncertainty (e.g. confidence intervals)
☐	☒	For null hypothesis testing, the test statistic (e.g. F, t, r) with confidence intervals, effect sizes, degrees of freedom and P value noted<br>*Give P values as exact values whenever suitable.*
☒	☐	For Bayesian analysis, information on the choice of priors and Markov chain Monte Carlo settings
☒	☐	For hierarchical and complex designs, identification of the appropriate level for tests and full reporting of outcomes
☐	☒	Estimates of effect sizes (e.g. Cohen's d, Pearson's r), indicating how they were calculated

Our web collection on statistics for biologists contains articles on many of the points above.

Software and code

Policy information about availability of computer code

Data collection	Widefield fuorescence images: EVOS FL Auto 2 Software (Life Technologies); confocal microscopy: ZEN Black 2.3; Flow cytometry: Attune NxT software 3.1.2 (Invitrogen)
Data analysis	Kallisto 0.48.0, sleuth 0.30.0, bwa 0.7.17, samtools 1.15.1, macs2 2.2.7, DESeq2 1.34.0, CIBERSORTx, samtools 1.15.1, Picard tools, SICER2, deeptools 2.2.0, bedtools 2.30.0, HOMER 4.11, ggplot2 3.3.6, pheatmap 1.0.12, Signac 1.8.0, CellAssign 0.99.2, inferCNV 1.13.0, REdiscoverTE 1.0, FiJi 2.9.0 (ImageJ), GraphPad Prism 9; R 4.1.1, FlowJo 9.0

For manuscripts utilizing custom algorithms or software that are central to the research but not yet described in published literature, software must be made available to editors and reviewers. We strongly encourage code deposition in a community repository (e.g. GitHub). See the Nature Portfolio guidelines for submitting code & software for further information.

Data

Policy information about availability of data

All manuscripts must include a data availability statement. This statement should provide the following information, where applicable:

- Accession codes, unique identifiers, or web links for publicly available datasets
- A description of any restrictions on data availability
- For clinical datasets or third party data, please ensure that the statement adheres to our policy

All RNA-seq,ATAC-seq and ChIP-seq datasets used in this study have been deposited to the Gene Expression Omnibus (GEO) with series IDs GSE149303, GSE149324 and GSE149334, and GSE212086. The scATAC-seq data of adult GBM has been previously published and is available in GEO (accession: GSE139136).

The scATAC-seq data of adult GBM has been published and is available in GEO (accession: GSE139136[https://www.ncbi.nlm.nih.gov/geo/query/acc.cgi?acc=GSE139136]). Survival data was previously published and is accessible via the r2 data portal[https://hgserver1.amc.nl/cgi-bin/r2/main.cgi]. The single-cell GBM data used in this study was previously published and is available at GSE131918[https://www.ncbi.nlm.nih.gov/geo/query/acc.cgi?acc=GSE131928] and on the Broad Single Cell Portal [https://singlecell.broadinstitute.org/single_cell/study/SCP503/gradient-of-developmental-and-injury-reponse-transcriptional-states-define-functional-vulnerabilities-underpinning-glioblastoma-heterogeneity]. Data on MACROH2A2 expression in mouse and human brain has been published and is available at Brain RNA-seq[https://www.brainrnaseq.org/] and DropViz [http://dropviz.org/]. Analysis code is available upon request. Data on MACROH2A2 expression in xenografts and multiregional samples was obtained from GSE139261[https://www.ncbi.nlm.nih.gov/geo/query/acc.cgi?acc=GSE139261] and GSE117891[https://www.ncbi.nlm.nih.gov/geo/query/acc.cgi?acc=GSE117891] respectively.

Human research participants

Policy information about studies involving human research participants and Sex and Gender in Research.

Reporting on sex and gender	Information on patient sex was collected as part of the tumour banking process, and gender was not considered. Sex-based analysis was not performed as analysis of cohorts showed no differences in macroH2A2 expression between male and female patients.
Population characteristics	Cell lines were obtained from 3 adult male patients with IDH-wildtype glioblastoma. Immunohistochemistry on primary samples was performed on 3 additional patient samples, of which 2 were male and 1 female. Ages ranged from 50 - 73 years.
Recruitment	Samples were obtained based on availability.
Ethics oversight	All samples were obtained from the University of Calgary Brain Tumour Tissue Bank in patients who had consented to tumour banking. Generation of the G523 cell line was performed at the Hospital for Sick Children (Toronto, ON) and approved by the institutional ethics board at that site. The tumour bank and present research project were all approved by the University of Calgary Conjoint Health Research Ethics Board (CHREB). Ethics protocol: HREBA.CC-16-0823.

Note that full information on the approval of the study protocol must also be provided in the manuscript.

Field-specific reporting

Please select the one below that is the best fit for your research. If you are not sure, read the appropriate sections before making your selection.

[x] Life sciences [ ] Behavioural & social sciences [ ] Ecological, evolutionary & environmental sciences

For a reference copy of the document with all sections, see nature.com/documents/nr-reporting-summary-flat.pdf

Life sciences study design

All studies must disclose on these points even when the disclosure is negative.

Sample size	No statistical methods were used to predetermine sample size. For animal experiments, we used 10 animals per group. For all sequencing experiments, we opted for at least 2 and typically 3 biological replicates. For in vitro experiments, we used at least 1 cell line and replicated multiple experiments in 2 additional lines. Sample sizes were selected to be sufficient to statistically validate hypotheses and were based on numbers typically used in our field and our group's previous studies.
Data exclusions	No data was excluded.
Replication	All major experiments were performed in biological duplicate or triplicate, and many of the findings in G523 were reproduced in knockdown lines of GSC2 and GSC3. Limiting dilution assays were assessed independently by two observers. All other experiments were performed at least once, and in biological duplicate or triplicate.
Randomization	Animal experiments: mice were randomly selected for injections into different groups. If injections were performed over multiple days, an equal number of animals from each group was injected on each day. Otherwise no randomization was performed.<br>In vitro experiments: no randomization was performed as the experimental protocols were standardized enough that systematic variability was minimized.
Blinding	For the in vivo mouse orthotopic xenograft limiting dilution experiment, team members were blinded to mouse group allocation. No blinding was performed for analysis of xenografts or any of the other experimental or computational studies, as analyses were performed using automated or computational methods and thus outcomes were less prone to observer bias.

Reporting for specific materials, systems and methods

We require information from authors about some types of materials, experimental systems and methods used in many studies. Here, indicate whether each material, system or method listed is relevant to your study. If you are not sure if a list item applies to your research, read the appropriate section before selecting a response.

Materials & experimental systems

n/a	Involved in the study
☐	☒ Antibodies
☐	☒ Eukaryotic cell lines
☒	☐ Palaeontology and archaeology
☐	☒ Animals and other organisms
☐	☒ Clinical data
☒	☐ Dual use research of concern

Methods

n/a	Involved in the study
☐	☒ ChIP-seq
☐	☒ Flow cytometry
☒	☐ MRI-based neuroimaging

Antibodies

Antibodies used	Rabbit anti-mH2A2 [1:250 ICC, WB], Novus NBP1-92094 [1:250 ICC;WB]; Rabbit anti-mH2A1 Millipore ABE215 [1:500 WB]; Goat anti-rabbit IgG HRP, Abcam ab6721 [1:20000 WB; 1:1000 IHC-P]; Invitrogen anti-mouse IgG Alexa Fluor 568 (A11011) [1:500 ICC], Invitrogen anti-rabbit Alexa Fluor 647 (A31573) [1:500 ICC], human anti-CD44 (Miltenyi Biotec 130-113-338) [1:100 flow cytometry; ICC] , Rabbit anti-Ki-67 (abcam ab 16667) [1:500 ICC], mouse anti-human nuclei (Millipore MAB1281) [1:200 ICC], Rabbit anti-OLIG2 (Millipore AB9610) [1:500 ICC,WB], Mouse anti-J2 antibody (Scicons #10010500) [1:200 ICC], Rabbit anti-FLAG antibody (Cell Signalling Technologies #14793) [1:500 ICC; 8 uL ChIP], Rabbit anti-mH2A2 (Invitrogen PA5-57437) [1:250 ICC, WB], mouse anti-beta-actin (Sigma A5441) [1:500 WB], rabbit anti-PDGFRA (Cell Signalling Technologies #3164) [1:500 WB], goat anti-mouse IgG-HRP (Abcam #6789) [1:2000 WB], mouse anti-SOX2 (Cell Signalling Technologies #2748) [1:500 IHC-P], mouse anti-Ki-67 (BD #550609) [1:200 ICC], rabbit anti-ASCL1 (CST #55467) [1:100 ICC], rabbit anti-lamin A/C (Abcam ab108595) [1:1000 WB], rabbit anti-H3 (CST #9715) [1:500 WB], mouse anti-GFAP (Millipore MAB360) [1:500 WB].
Validation	The anti-macroH2A2 antibodies were additionally validated by Western blot of macroH2A2 knockdown lines. Immunohistochemical stains were validated by blotting versus IgG and secondary-only controls. ChIP for FLAG antibody was validated by ChIP against rabbit IgG. All antibodies were validated for the applications used as per the specifications listed by the manufacturer websites and have been cited in other previous publications.

Eukaryotic cell lines

Policy information about cell lines and Sex and Gender in Research

Cell line source(s)	Cell lines were obtained from the lab of Dr. Peter Dirks (G523, derived by the Dirks lab; male patient) or generated in house from primary patient samples (GSC2 - male patient, GSC3 - male patient). All were primary cell lines derived from primary adult glioblastoma patients.
Authentication	Cell lines were authenticated using STR genotyping.
Mycoplasma contamination	All cells lines used in this study were tested for mycoplasma contamination using the Lonza MycoAlert kit and were found to be negative.
Commonly misidentified lines (See ICLAC register)	None were used.

Animals and other research organisms

Policy information about studies involving animals; ARRIVE guidelines recommended for reporting animal research, and Sex and Gender in Research

Laboratory animals	All animals used in this study were female NOD-SCID-IL2R-gamma-null mice (JAX #5557) at 3 months of age. Animal protocol ID: AC21-0226. Animals were housed and bred in a specific pathogen-free animal facility under standard conditions. Housing conditions were as follows: ambient temperature of 21-23 C with low humidity, 12 hr light-dark cycles with light from 7 am - 7 pm.
Wild animals	No wild animals were used in this study.
Reporting on sex	All animals used were female, and as such no sex-based analysis was performed.
Field-collected samples	The study did not involve samples collected from the field.
Ethics oversight	The animal protocols were approved by the University of Calgary Conjoint Health Research Ethics Board (CHREB) and the Animal Care Committee at the University of Calgary (protocol ID AC21-0226) and all institutional regulations were followed.

Note that full information on the approval of the study protocol must also be provided in the manuscript.

Clinical data

Policy information about clinical studies

All manuscripts should comply with the ICMJEguidelines for publication of clinical research and a completedCONSORT checklist must be included with all submissions.

Clinical trial registration	*Provide the trial registration number from ClinicalTrials.gov or an equivalent agency.*
Study protocol	*Note where the full trial protocol can be accessed OR if not available, explain why.*
Data collection	*Describe the settings and locales of data collection, noting the time periods of recruitment and data collection.*
Outcomes	*Describe how you pre-defined primary and secondary outcome measures and how you assessed these measures.*

ChIP-seq

Data deposition

- [x] Confirm that both raw and final processed data have been deposited in a public database such as GEO.
- [x] Confirm that you have deposited or provided access to graph files (e.g. BED files) for the called peaks.

Data access links<br>*May remain private before publication.*	GSE212086
Files in database submission	fastq files, bed files of consensus peak regions
Genome browser session<br>(e.g. UCSC)	Not applicable

Methodology

Replicates	Three biological replicates were sequenced alongside an input sample.
Sequencing depth	Sequencing was performed using 2x100 paired end reads (NovaSeq SP200 cartridge). All samples were sequenced to a minimum depth of 193,000,000 reads, of which 85-99% consisted of properly paired mate reads.
Antibodies	rabbit anti-FLAG antibody (Cell Signalling Technologies #14793)
Peak calling parameters	Peaks were called using SICER2 using default parameters, an hg38 reference, and input as control.
Data quality	ChIP-seq was assessed using the ENCODE quality standards and additional quality control was performed using deeptools 2.2.0, using the tools multiBamSummary, and plotCorrelation (using a spearman correlation), as well as plotFingerprint.
Software	Samples were aligned using bwa 0.7.17 with the mem algorithm to the GRCh38 (hg38) human reference genome. Additional filtering was applied using samtools to remove reads mapping to blacklist regions and reads with q < 30. Duplicates were marked and removed using Picard. Peaks were called using SICER2 with default parameters and using an input sample as reference. Peaks from different replicates were merged to create a consensus peaklist using bedtools merge. Pileups for visualization were generated using the deeptools command bamCompare, using the settings -bs 20, -smoothLength 100 --operation ratio. Permutation testing was performed using regioneR, as for ATAC-seq. Motif calling and gene ontology analysis was performed using HOMER.

Flow Cytometry

Plots

Confirm that:

- [x] The axis labels state the marker and fluorochrome used (e.g. CD4-FITC).
- [x] The axis scales are clearly visible. Include numbers along axes only for bottom left plot of group (a 'group' is an analysis of identical markers).
- [x] All plots are contour plots with outliers or pseudocolor plots.
- [x] A numerical value for number of cells or percentage (with statistics) is provided.

Methodology

Sample preparation	Glioma cells grown on PLO-laminin plates were passaged with Accutase (Stem Cell Technologies) and resuspended in PBS. Cells were stained using an APC-conjugated anti-human anti-CD44 antibody (REA690; 1:200 concentration) for 20 minutes at 4 C on a rotator. Cells were washed twice in PBS, and passed through a cell strainer top tube to remove any clumps (Corning 352235). Cells were stained at 1:1000 with Dye Cycle Violet (Life Technologies V35003) and incubated at 37 C for 30 minutes, as per manufacturer protocols.

Instrument	Attune NxT flow cytometer (Invitrogen)
Software	Data was collected with the Attune NxT Flow Cytometer software and anaysed using FlowJo and CytoExploreR.
Cell population abundance	The purity of GFP-positive cells was approximately 80% in all experiments.
Gating strategy	Cells were first gated by FSC/SSC in order to exclude debris, then singlets were selected by gating on FSC-A and FSC-H. Following this, a GFP-positive population was gated using a fluoresence-negative cell-type matched control.

☒ Tick this box to confirm that a figure exemplifying the gating strategy is provided in the Supplementary Information.
