# Supplementary material for: macroH2A2 antagonizes epigenetic programs of stemness in glioblastoma
Source: Nat Commun. 2023 May 27;14:3062. doi: 10.1038/s41467-023-38919-2 (PMC10224928; doi:10.1038/s41467-023-38919-2)
Supplement: Supplementary file 3 — Description of Additional Supplementary Files [file 41467_2023_38919_MOESM3_ESM.pdf]

Title: Supplementary Data 1
Description: Summary of genomic analyses and primer sequences.
